# Diversification and Functional Evolution of HOX Proteins

**DOI:** 10.3389/fcell.2022.798812

**Published:** 2022-05-13

**Authors:** Narendra Pratap Singh, Robb Krumlauf

**Affiliations:** ^1^ Stowers Institute for Medical Research, Kansas City, MO, United States; ^2^ Department of Anatomy and Cell Biology, Kansas University Medical Center, Kansas City, KS, United States

**Keywords:** gene duplication and divergence, protein evolution, HOX proteins, *Drosophila*, mouse

## Abstract

Gene duplication and divergence is a major contributor to the generation of morphological diversity and the emergence of novel features in vertebrates during evolution. The availability of sequenced genomes has facilitated our understanding of the evolution of genes and regulatory elements. However, progress in understanding conservation and divergence in the function of proteins has been slow and mainly assessed by comparing protein sequences in combination with *in vitro* analyses. These approaches help to classify proteins into different families and sub-families, such as distinct types of transcription factors, but how protein function varies within a gene family is less well understood. Some studies have explored the functional evolution of closely related proteins and important insights have begun to emerge. In this review, we will provide a general overview of gene duplication and functional divergence and then focus on the functional evolution of HOX proteins to illustrate evolutionary changes underlying diversification and their role in animal evolution.

## Introduction

**FIGURE 1:**
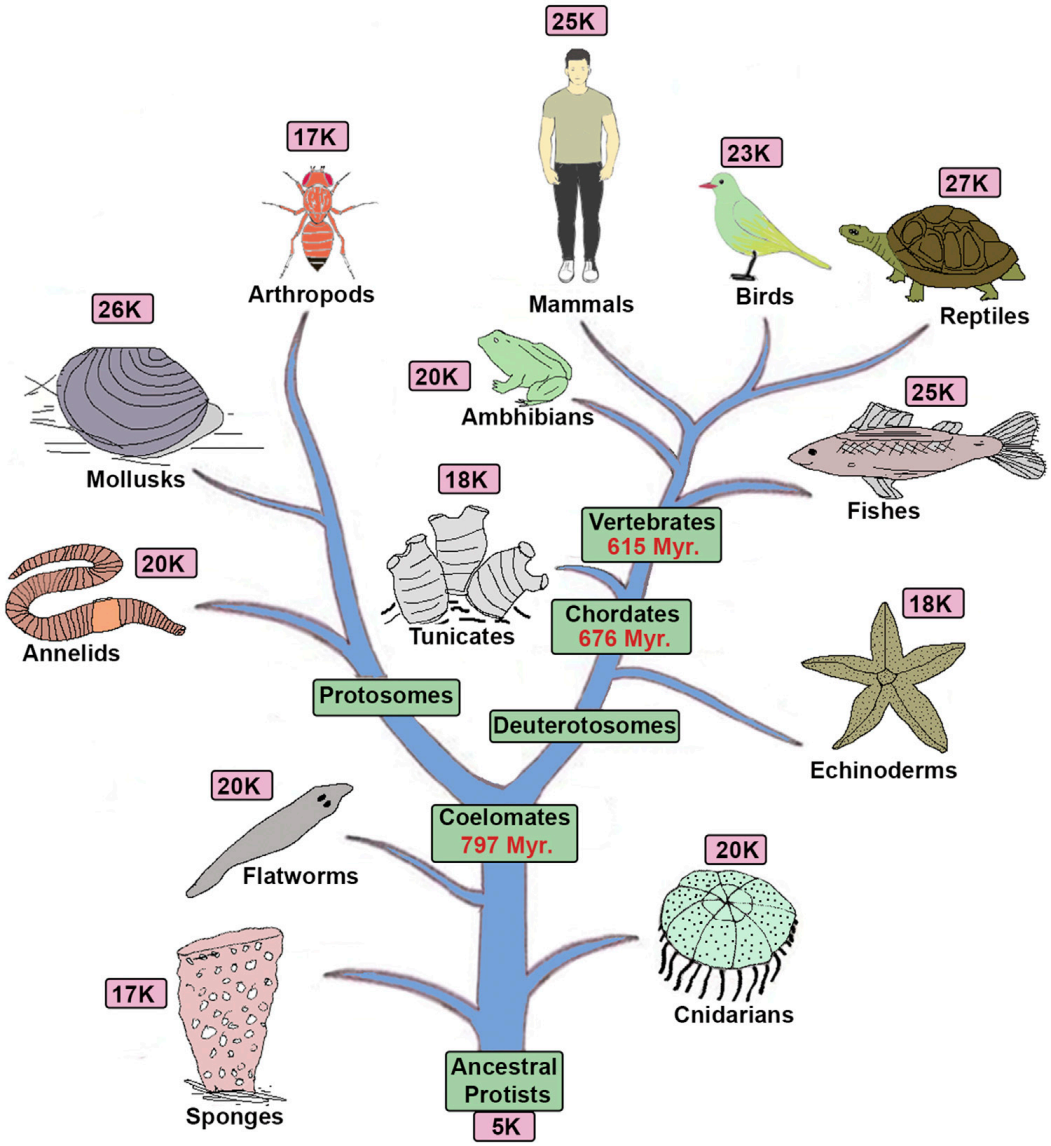
Tree of life and gene complexity: The diagram shows a phylogenetic tree of animals with the approximate total number of genes present in each genome based on sequencing of their representative genomes (Hedges et al., 2015).

Evolution has brought an incredible range of morphological and physiological novelties to diverse animals. Centuries of classical research has served to catalog diverse novelties in 1.2 million species and sub-divide them into ∼36 phyla and 107 classes, 500 orders, 5500 families and 110000 genera (Mora et al., 2011). These efforts have uncovered the emergence of novelties during the progressive evolution of animals, but we know relatively little about the genetic and genomic changes and mechanisms that underlie this diversity. Technological advances which enabled the systematic sequencing of animal genomes has reenergized this field of research and provided an opportunity for comparative genomics of the diverse animals to probe the underlying genetic causes of their morphological and physiological differences (Rogers and Gibbs, 2014). These genome-wide analyses have highlighted common origins and similar physiological functions but have found it challenging to uncover the genetic changes and mechanisms that underlie animal diversity. Comparative genomic analyses reveal a very similar number of genes in diverse animals (Figure 1), indicating that the total gene number does not reflect diversity (Hahn and Wray, 2002; Copley, 2008). Furthermore, many of the same genes and gene families are present in a broad range of animal species, suggesting there is a shared or common “gene toolkit.”

The discovery of a very similar number of genes and a common “gene toolkit” in animal genomes appeared to refute the hypothesis that gene duplication and diversification is a major contributor to animal diversity. This led to a shift in the focus of research from analyses of coding regions to identifying and characterizing diversification of *cis*-regulatory (non-coding) regions and gene regulatory networks embedded in the genome (Biemont and Vieira, 2006). A broad array of technological advances have also revolutionized this field and enhanced our ability to identify and functionally validate the *cis*-regulatory code embedded in the genome by the integration of comparative genomics, transgenic analyses, CRISPR/Cas9 genome modifications and genome-wide approaches (i.e., ChIP-seq, ATAC-seq, massive parallel reporter assays and single cell transcriptomics) (Zhen and Andolfatto, 2012; Paul et al., 2014; He et al., 2015; Gasperskaja and Kucinskas, 2017; Avsec et al., 2021). Application of these approaches, has revealed that diversity in non-coding *cis*-regulatory regions of the genome has played a major role in the emergence of animal diversity (Carroll, 2008; Rubinstein and de Souza, 2013; Reilly and Noonan, 2016; Franchini and Pollard, 2017; Xie et al., 2019; Roberts Kingman et al., 2021a; Roberts Kingman et al., 2021b).

Evaluating how changes in protein sequence impact *in vivo* function following gene duplications in animal evolution has been challenging to investigate in the absence of technologies for precise manipulation of endogenous genes and quantitative and qualitative functional assays to evaluate activity in an *in vivo* context. The development of CRISPER/Cas9 gene editing technology for precise manipulation of endogenous genes in the genome of diverse animals has opened the door for more direct cross-species comparisons of homologous protein functions (Doudna and Charpentier, 2014). This approach has the advantage of expressing the proteins being compared at the same physiological levels in their normal spatial, temporal and tissue-specific contexts under control of regulatory components of the endogenous loci. In the past, functional studies have primarily relied on comparing the degree of conservation in amino acid sequences, *in silico* structure predictions, *in vitro* assays for activity and ectopic over-expression assays *in vivo*. The *in vitro* assays of protein activity, such as ligand binding, enzymatic activity, transcription factor binding properties, can be limited by *ex-vivo* conditions, which often lack key co-factors or components important *in vivo*. Hence, they may provide a limited perspective on a subset of functional activities relevant to their *in vivo* roles. To overcome some of the limitations of *in vitro* assays, transgenic approaches have been used to ectopically express genes in the animals and compare the *in vivo* properties of candidate proteins (Mcginnis et al., 1990; Quiring et al., 1994; Hanks et al., 1998). However, this approach often involved broad over-expression of proteins at high levels and ectopic sites, making it difficult to compare activities in normal physiological and developmental contexts. CRISPR/Cas9 technologies now offer possibilities to manipulate the genome to precisely compare function of homologous genes *in vivo*. As a result, cross-species analyses of gene function are beginning to uncover unexpected changes and mechanisms that contribute to conservation and divergence of protein functions contributing to animal diversity (Enard et al., 2009; Truong and Boeke, 2017; Laurent et al., 2020; Singh et al., 2020). In addition, recent advances in cryo-EM and computational approaches for predicting protein structures are rapidly changing our ability to analyze and compare the properties of proteins (Assaiya et al., 2021; Jumper et al., 2021).

## Gene Duplication and Divergence

Sequence analyses have revealed a high level of conservation of many domains in proteins with very diverse functions across the animal phylum (Laity et al., 2001; Ponting and Russell, 2002; Noyes et al., 2008). This implies that during evolution, the appearance of novel functions is not associated with widespread *de novo* evolution of new genes and that novel functional activities most likely arose by diversification of existing genes (Holland et al., 1994; Hughes, 1994; Friedman and Hughes, 2001; Blomme et al., 2006). Altering the function of an essential gene could be detrimental to the survival or fitness of a species, but gene duplication events provide a mechanism to circumvent this limitation. Generating multiple copies of a gene provides a range of opportunities to maintain essential functions, releasing selective pressure on a single essential gene, while also producing new substrates that can diversify and evolve novel functions. Analyses of gene sets across the animal kingdom revealed that vertebrate genomes have multiple copies of many invertebrate genes, including those that regulate development, differentiation and physiological processes, such as transcription factors, cell signaling pathways, odorant receptor genes etc. (Paps and Holland, 2018; Richter et al., 2018; Fernandez and Gabaldon, 2020). Large genome duplication events followed by gene losses are considered as a critical step in the emergence and evolution of vertebrates. Susumu Ohno suggested that two rounds of whole-genome duplications (2R-WGDs) could be a major source of gene amplification and functional diversification in vertebrate lineage (Ohno, 1970; Panopoulou et al., 2003; Vandepoele et al., 2004; Dehal and Boore, 2005). While this hypothesis was used to explain a major cause of gene duplications, comparing all homologous gene families between *Drosophila* and humans showed that less than 5% of these families display a predicted 1:4 gene ratio (Friedman and Hughes, 2001). This was not consistent with Ohno’s hypothesis and lead to an alternative idea, suggesting that selective regional duplications (segmental duplications) created a mixed repertoire of duplicated genes in the genome.

**FIGURE 2:**
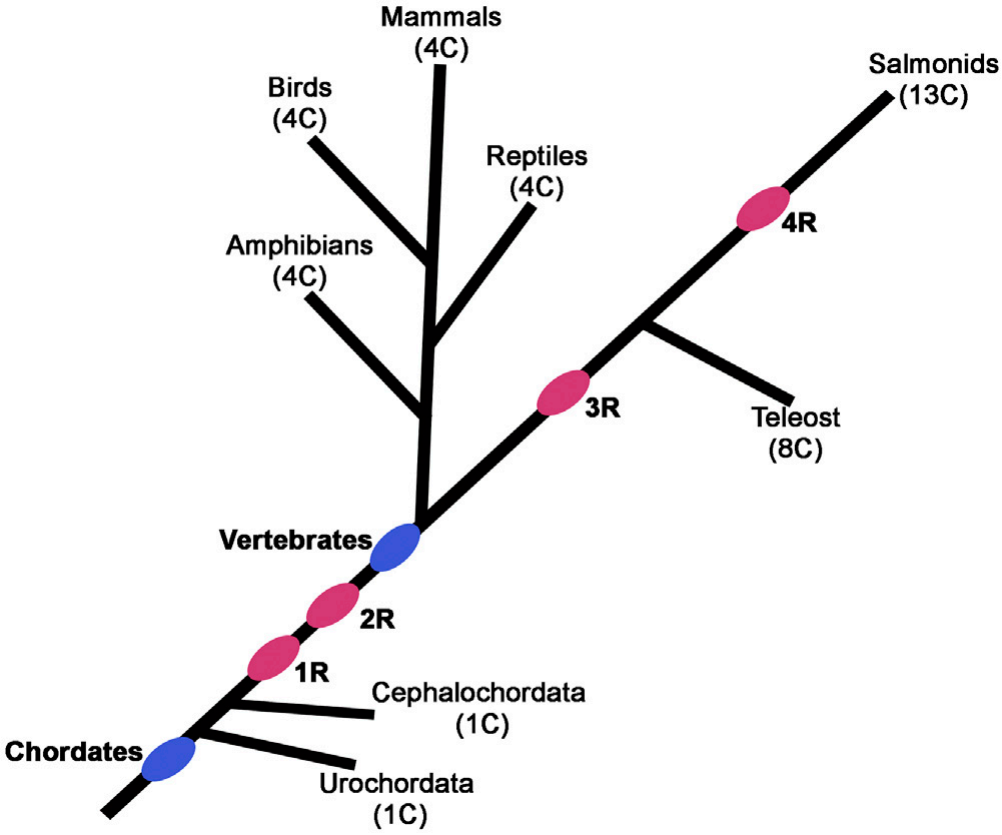
Phylogenetic tree depicting the position of postulated whole-genome duplication (R) events during vertebrate evolution. The pink ovals indicate the progressive rounds of whole-genome duplication. The number of Hox clusters (C) in the different species is indicated in parentheses.

Despite these conflicting models, which remain challenging to resolve, there is evidence for whole genome duplications in many plants, yeast, and vertebrate species, such as teleost fish (3R), salmonid fish (4R) and *Xenopus laevis* (Wendel, 2000; De Bodt et al., 2005; Scannell et al., 2006; Pascual-Anaya et al., 2013; Session et al., 2016) (Figure 2). Advocates of the 2R hypothesis have further refined the model to suggest that during vertebrate evolution frequent gene loss after duplication, as a consequence of redundancy, contributed to the observed digression from the expected 1:4 gene ratio. Analyses of many known genome duplications indicate that gene loss is the most common fate of duplicated genes (Nadeau and Sankoff, 1997; Albalat and Canestro, 2016). Loss of many duplicated genes and nonessential genes could also explain how a similar number of total genes are present in higher animal genomes despite differences in the whole genome or segmental duplication events. Despite the controversy on the underlying mechanisms for widespread amplification of gene families (Braasch et al., 2018; Sandve et al., 2018), the importance of gene duplication in creating a major substrate for functional divergence and emergence of novel functions is widely accepted in the field (Lynch and Conery, 2000).

## Fate of the Duplicated Genes

The contribution of gene duplication and divergence to the emergence of novel protein functions in animal evolution has gained more traction as gene functions have been characterized in different animals across the phylogenetic spectrum (Blomme et al., 2006). Comparing classes of genes based on their functions revealed that genes involved in animal development and cell signaling pathways are highly amplified during the evolution, while many other classes of genes have been lost. Based on these analyses several hypotheses came to explain the forces that shaped the future of a duplicated genes. Loss of function is the most common fate of the duplicated genes. Studies have shown that only half of duplicated genes are retained while others lose functional activities by processes that include deletion, rearrangement, point mutations, and pseudogene formation (Nadeau and Sankoff, 1997; Blomme et al., 2006; Scannell et al., 2006; Albalat and Canestro, 2016; Guijarro-Clarke et al., 2020a; b). Even in the absence of duplicated genes, a large number of genes (90% in bacteria, 80% in yeast, 65% in *C. elegans* and 85% in *Drosophila*) are dispensable for animal survival, which provides a large set of substrates for evolutionary change. Gene duplication events further expand the repertoire of substrates for change and generate opportunities for functional redundancy that allows for non-deleterious functional diversification of genes.

Essential genes and their functional roles need to be retained to ensure animal survival and fitness. Hence, at least one member of a duplicated gene family must retain the key ancestral functions during evolution. Other members are free to accumulate mutations that potentiates diversification and the emergence of novel activities, which is called neofunctionalization (Ohno, 1970; Clark, 1994; Holland et al., 1994; Lundin, 1999; Friedman and Hughes, 2001; Mazet and Shimeld, 2002; Sandve et al., 2018). A variation of this idea is that the ancestral functions of essential genes maybe collectively retained by partitioning sub-sets of the functional roles between different duplicated family members, which is termed subfunctionalization (Ohno, 1970; Force et al., 1999; Lynch and Force, 2000; Sandve et al., 2018). These are not mutually exclusive processes. A study by Le and Zhang demonstrated that neofunctionalization or subfunctionalization alone do not adequately explain the diversification of protein function. They proposed that many duplicated genes may go through a combination of subfunctionalization and neofunctionalization to produce duplicated genes that possess new and retain some ancestral roles (He and Zhang, 2005; Marcussen et al., 2010). Various theoretical and functional studies have explored these ideas and confirmed that the retention of duplicated genes appears to be mediated by a varying combination of these processes (Force et al., 1999; Lynch and Force, 2000; Vandenbussche et al., 2003; Walsh, 2003; Burki and Kaessmann, 2004; Escriva et al., 2006; Perry et al., 2007; Kleinjan et al., 2008; Innan, 2009; Truong and Boeke, 2017; Zimmer et al., 2018; Singh et al., 2020).

## *Hox* Genes as a Paradigm for Duplication and Divergence of Function

**FIGURE 3:**
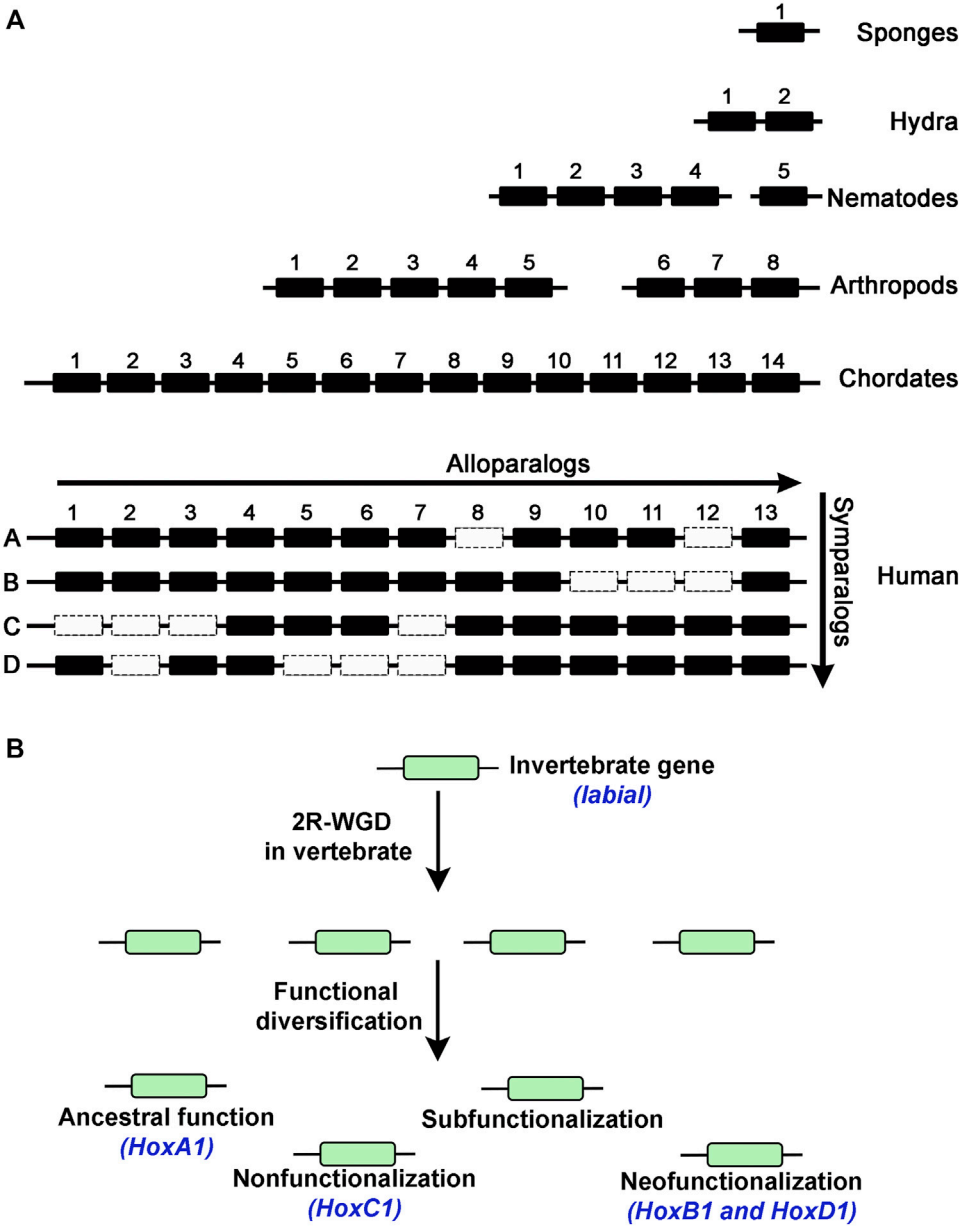
**(A)** Diagram shows number of *Hox* genes in different animals indicating expansion of the Hox cluster into alloparalogs. Duplication events in vertebrates generated 4 Hox clusters to create symparalogs, Human genome has 39 *Hox* genes suggesting loss of some genes (dotted box) during evolution. **(B)** Possibilities of functional diversification of duplicated genes in the vertebrate genome after duplication. An example of the invertebrate *labial* gene from *Drosophila*, duplication in vertebrates and functional diversification of the homologous *Hox1* genes (blue text).

*Hox* genes, encode a broadly conserved family of transcription factors in animals, and represent an interesting paradigm for examining the duplication and divergence of gene functions. The HOX proteins are involved in patterning and specification of the anterior-posterior (AP) axis of all bilaterian animals (Mcginnis and Krumlauf, 1992; Krumlauf, 1994; Carroll, 1995; Pearson et al., 2005). The temporal and spatial order of *Hox* gene expression and function across the embryo is “colinear” and correlated with their organization along the chromosome (Lewis, 1978; Duboule and Dolle, 1989; Graham et al., 1989; Duboule, 1998; Kmita and Duboule, 2003). These genes are typically found to be tightly clustered in the genome except for some animals where evolution has led to the disintegration of the ancestral complex (Kaufman et al., 1980; Akam et al., 1994; Seo et al., 2004; Sekigami et al., 2017). Each gene in a cluster specifies distinct cellular identities along AP axis during very early embryonic development, which ultimately patterns tissues and structures in adult animals. Evidence of *Hox* genes in animal genomes is traced back to Cnidarians, however, their role in patterning the AP axis is observed only in bilaterians, as they have roles in patterning radial segmentation in cnidarians (Pascual-Anaya et al., 2013; Arendt, 2018; He et al., 2018). Mutations that affect the expression and function of *Hox* genes in bilaterians lead to homeotic transformation of one part of the body into another (Lewis, 1994). Furthermore, diversification of *Hox* gene number and function correlates with increased diversity in the evolution of animals (Wagner et al., 2003; Lemons and Mcginnis, 2006). There are fewer *Hox* genes in lower invertebrates as compared to higher invertebrates, chordates, and vertebrates, as illustrated by the 5 *Hox* genes in nematodes (*C. elegans*), 8 in arthropods (*Drosophila*), 14 in chordates, and 39 in mammals (Human) (Ikuta, 2011; Pascual-Anaya et al., 2013; Irie et al., 2018) (Figure 3A).

Despite their diverse and distinct functional activities, sequence analysis indicates that *Hox* gene cluster was generated by tandem duplication of a single gene. Analysis of *Hox* gene clusters in invertebrate genomes suggests that they expanded from a common ancestral gene through tandem regional duplication events to form a maximum of fifteen alloparalogs in cephalochordates (ex. *Branchiostoma floridae)* (Garcia-Fernandez and Holland, 1994; Koonin, 2005). There are four Hox clusters in mammalian genomes with a maximum of 14 genes in each cluster suggesting two rounds of whole complex duplication (2R) from a common invertebrate ancestor with 14 genes (Maconochie et al., 1996; Hoegg and Meyer, 2005; Lemons and Mcginnis, 2006; Duboule, 2007; Kuraku and Meyer, 2009; Pascual-Anaya et al., 2013; Holland and Ocampo Daza, 2018; Smith et al., 2018). However, there is a maximum of 13 *Hox* genes in each cluster of tetrapod genomes, such as mouse and human, indicating a loss of 14th paralog during vertebrate diversification (Figure 3A). There is also evidence for two additional lineage specific whole-genome duplications events in vertebrates, one in teleosts (3R) (Pascual-Anaya et al., 2013) and an additional round (4R) in salmonid lineages to further amplify HOX genes (Figure 2) (Soshnikova et al., 2013; Vieux-Rochas et al., 2013). These data suggest that genome duplication events have dramatically increased the number of *Hox* genes in vertebrate genomes and provide opportunities for evolution of novel functions. Further, functional compensation or redundancy after gene duplication events allowed for loss and diversification of these genes which have a critical role in patterning the AP axis and the properties of tissues in a manner that has been remarkably conserved across the bilateral animals (Wagner et al., 2003).

## Conservation and Diversification of *Hox* Gene Function

The correlation between the expansion of *Hox* genes, a master regulator of development, and greater complexity of vertebrates highlights the importance of studying the functional evolution of HOX proteins. There are reports to suggest both conservation and diversification of *Hox* gene function through evolution (Lawrence and Morata, 1994; Saurin et al., 2018). Early work in the field has demonstrated that HOX proteins from different alloparalogs groups defines distinct morphological features along the AP body axis implying that each regulates a distinct set of gene regulatory networks and developmental pathways (Garcia-Bellido et al., 1973; Mcginnis and Krumlauf, 1992; Lawrence and Morata, 1994; Averof, 2002; Maeda and Karch, 2006). However, HOX proteins appear to share many similar biochemical properties. They display nearly identical DNA binding specificities *in vitro*, as a consequence of the presence of a highly conserved 60 amino acid long homeodomain (HD) (Scott et al., 1989; Gehring et al., 1994a; Piper et al., 1999). HOX proteins have the ability to interact with a PBC class (e.g., PBX, MEIS, PREP) of homeodomain transcription factors, which serve as cofactors in binding DNA and modulating gene expression (Desplan et al., 1988; Gehring et al., 1994a; Knoepfler and Kamps, 1995; Mann and Chan, 1996; Noyes et al., 2008). The interaction of HOX proteins with PBC factors is primarily mediated by a conserved six amino acid domain, referred to as the hexapeptide (HP). HOX-PBX cofactor interactions modify the affinity and specificity of HOX DNA binding on target sites in the genome (Slattery et al., 2011; Merabet and Mann, 2016). These generic DNA binding properties of HOX proteins and interactions with shared cofactors, such as PBX, make it difficult to explain the paralog-specific functions of HOX proteins. Hence, the distinct differences in the functional roles and genome-wide binding preferences of HOX proteins *in vivo* is likely to be a consequence of additional unidentified features and interactions of HOX proteins that impact and modulate their context-dependent activities.

### Conservation

Early studies on *Hox* genes explored the evolutionary conservation of functions among homologous genes across the animal evolution by using transgenesis to express vertebrate *Hox* genes in *Drosophila* (Malicki et al., 1990; Mcginnis et al., 1990; Zhao et al., 1993; Lutz et al., 1996). These studies uncovered deep conservation of *Hox* gene function in specifying regional identity along the AP body axis over 600 million years of animal evolution. Protein sequences of alloparalogs, formed by tandem duplication of the ancestral *Hox* genes, show high diversification and each of them are known to drive distinct developmental and differentiation programs to regulate regional identities in specific tissues along the AP axis (Lamka et al., 1992; Mclain et al., 1992; Yokouchi et al., 1995; Carapuco et al., 2005). Despite these differences in functional roles, many alloparalogs show redundancy or overlaps of function in regulating developmental events in some specific tissues (Saurin et al., 2018). For example, it has been observed that ubiquitous expression of many *Hox* genes is important in *Drosophila* larval fat body cells, which appears distinctly different from their roles along the AP axis facilitated by their nested collinear expression patterns in embryos. Furthermore, functional analyses in autophagy inhibition uncovered no paralog specificity and suggested redundant functions of many HOX proteins (Banreti et al., 2014). Similarly, *Drosophila Hox* genes have been also shown to have redundant activity in specification of tritocerebrum identity, endocrine ring gland development, dorsal DA3 muscle lineage specification and head repression etc. (Hirth et al., 2001; Coiffier et al., 2008; Enriquez et al., 2010; Sanchez-Higueras et al., 2014). The redundant role of *Hox* genes is not limited to *Drosophila*, vertebrate HOX proteins have also been shown to have overlapping or redundant functions during development of several tissue types (Young et al., 2009; Lacombe et al., 2013; Denans et al., 2015). For example, vertebrate *Hox6* paralogs (*Hoxa6*, *Hoxc6*, and *Hoxb6*) are required to specify lateral motor column motoneurons and functional studies displayed that Hox paralogs 5, 7, and 8 can all substitute for this function (Lacombe et al., 2013). These observations suggest that despite evidence for sequence and functional diversification among HOX alloparalogs, which underlies their distinct roles in axial patterning, some aspects of their functional activities have been conserved during evolution and play roles in specific tissue contexts during development.

As expected, the functional redundancy among the *Hox* genes is more common among the symparalogs formed more recently after whole cluster duplications in vertebrate lineage (Figure 3A). Deletion of a single gene or even a whole cluster does not show dramatic consequences on embryonic development, consistent with the idea of extensive functional redundancy between *Hox* genes (Medina-Martinez et al., 2000; Suemori and Noguchi, 2000; Spitz et al., 2001; van Den Akker et al., 2001; Hunter and Prince, 2002; Soshnikova et al., 2013). Gene swap experiments in mouse models have also demonstrated that symparalogs, formed after duplication of ancestral invertebrate cluster, are functionally equivalent (Horan et al., 1995; Manley and Capecchi, 1997; Greer et al., 2000; Tvrdik and Capecchi, 2006; Iacovino et al., 2009). An interesting example is a gene swap of mouse *Hoxa3* and *Hoxd3* that resulted in adult mice with no detectable developmental defects (Greer et al., 2000). These observations suggests that HOX paralogs have retained similarity in their activity through millions of years of evolution, which has been attributed to conservation of DNA binding properties of the homeodomain and a shared hexapeptide domain that mediates interaction with PBC factors.

### Diversification

Over expression studies have provided evidence that Hox alloparalogs regulate development of specific organs across the body axis, indicating that they can drive distinct gene regulatory networks (Schneuwly et al., 1987; Lamka et al., 1992; Mclain et al., 1992; Yokouchi et al., 1995; Carapuco et al., 2005). Comparative analyses of gene expression profiles upon ubiquitous expression of *Drosophila Hox* genes show a very small number (1.3%) of common changes, and most of changes are unique to each gene, suggesting they individually regulate distinct set of targets (Hueber et al., 2007). This data implies that despite the high conservation of homeodomain region, HOX proteins regulate distinct set of genes to specify unique cellular identities across the AP body axis during animal development (Mcginnis and Krumlauf, 1992; Averof and Akam, 1993; Lawrence and Morata, 1994; Patterson et al., 2001; Averof, 2002). The functional diversity among the HOX proteins may be generated in multiple ways. Domain swaps of the HD, the most conserved region of HOX proteins, can result in functionally distinct activity in some developmental contexts. This suggests that even a small number of changes can lead to diversification of the DNA binding properties and transcriptional activity of HOX proteins (Zhao and Potter, 2001; 2002). In addition, studies have shown that amino acid differences among the Hox alloparalogs may not alter DNA binding preference but change their ability to recruit different coactivators or corepressors (Li and Mcginnis, 1999; Gebelein et al., 2004; Joshi et al., 2010). Another mechanism for generating diversity among HOX proteins is through its interaction with PBC group of cofactors, which alters DNA binding specificity. In fact, a high throughput study revealed that DNA binding specificities of HOX-Exd complex are only revealed upon heterodimerization (Slattery et al., 2011). These observations suggest that HOX proteins are subject to a variety of ways that can diversify or modulate their functional properties.

**FIGURE 4:**
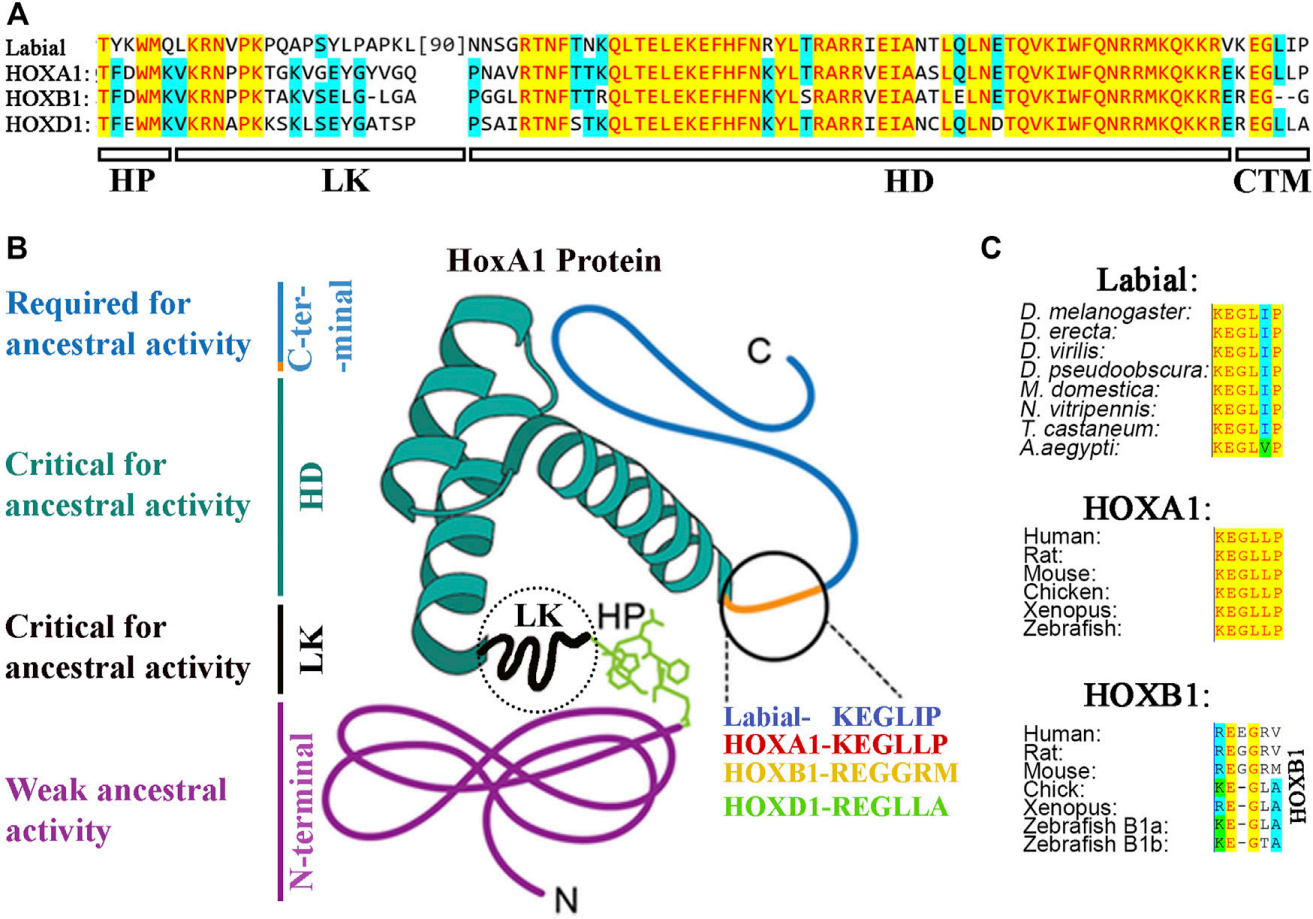
**(A)** Protein sequence alignment of hexapeptide (HP), linker (LK), Homeodomain (HD) and CTM region of Drosophila Labial with that of mouse HOX1 paralogs. **(B)** Distribution of ancestral activity in HOXA1 protein. **(C)** Alignment of CTM region in arthropod Labial, vertebrate HOXA1 and HOXB1.

As discussed earlier, there are many examples of functional redundancy among the HOX symparalogs (Horan et al., 1995; Chen and Capecchi, 1997; Greer et al., 2000; Wahba et al., 2001; Tvrdik and Capecchi, 2006; Iacovino et al., 2009). However, several studies have also found functional diversification among symparalogs (Fromental-Ramain et al., 1996; Miguez et al., 2012; Singh et al., 2020; Singh et al., 2021). Analyses of *Hoxa9* and *Hoxd9* mutants in mouse revealed that these two symparalogous genes have both specific and redundant functions in lumbosacral axial skeleton patterning and in limb morphogenesis (Fromental-Ramain et al., 1996). Similarly, *Hoxa2* and *Hoxb2* symparalogs show synergistic interactions in regulation of gene expression in the hindbrain, however, during oligodendrogenesis in the mouse hindbrain *Hoxb2* antagonizes *Hoxa2* function (Davenne et al., 1999; Miguez et al., 2012). These analyses indicate that symparalogs have retained a lot of overlapping functions during evolution, but they have also diversified their functional roles thought changes in the patterns of expression and protein structure. An important understudied question in the field is how changes in amino acid sequences of symparalogous after duplication from the ancestor homolog relate to altered functions. Analyses of HOX1 proteins in Xenopus suggests they have redundant roles of HOXA1, B1 and D1 in hindbrain development (Mcnulty et al., 2005). Gene swaps of HoxA1 and HoxB1 in mouse also suggest they have largely overlapping or redundant roles (Tvrdik and Capecchi, 2006). However, in a recent study, we utilized CRISPR/Cas9 technology to replace the *Drosophila Hox* gene *labial* with its mouse homologs *HoxA1*, *HoxB1* and *HoxD1* to investigate conservation of ancestral functions and assess diversification during evolution (Singh et al., 2020). Despite similar degrees of protein sequence diversification of mouse HOX1 proteins from *Drosophila* Labial, our results revealed that among the HOX1 symparalogs (HOXA1, HOXB1 and HOXD1) only *HOXA1* is able to rescue *labial* function, as HOXB1 and HOXD1 failed to do so (Figures 3B, 4A). This demonstrates a remarkable conservation of ancestral activity by HOXA1 and indicates that HOXB1 and HOXD1 have diversified through 600 million years of evolution (Figure 3B). Furthermore, consistent with their share ancestral activities, comparative genome-wide DNA binding properties revealed that HOXA1 and Labial have similar patterns of binding in mouse genome, while HOXB1 binds to a distinctly different set of targets. This adds support for neofunctionalization of HOXB1 by regulating a district gene regulatory program (Figure 3B) (Singh et al., 2020; Singh et al., 2021). Studies on mouse HOXD1 have shown that it has lost ancestral activities and appears to have undergone neofunctionalization through expression in new tissue types and altered activities in regulating novel gene regulatory programs (Guo et al., 2011). These distinct functional properties of mouse HOX1 symparalogs illustrate the diversification of HOXB1 and HOXD1 function and loss of ancestral activity in mammalian lineage. Mapping of the protein sequences that underlie functional diversification of HOXA1 and HOXB1 proteins revealed that a small number of changes across the protein cause functional diversification. It will be important to examine similar changes in other paralogy groups of HOX proteins to determine if this is a common means for modulating functional activities.

## Critical Regions Underly HOX Protein Function and Functional Diversification

The deep conservation of HOX protein function across bilaterians and their redundant role in some tissue types suggest that the essential roles of HOX proteins in AP patterning has restricted their diversification. Preservation of many of their ancestral functions is likely mediated through conservation of the homeodomain and hexapeptide regions. The homeodomain of *Drosophila* HOX proteins is known to bind directly to DNA (Galant et al., 2002; Merabet et al., 2003). However, on many *in vivo* target sites, binding affinity and specificity is enhanced by interaction with PBC factors (Loker et al., 2021). The regions outside these domains are highly diversified among the HOX proteins that can impact activation or repression of transcription of potential target genes (Li et al., 1999; Li and Mcginnis, 1999). In this section of the review, we will discuss evidences on what is known about various domains of HOX proteins and how they may have diversified to adopt novel functions during evolution.

### Homeodomain

**FIGURE 5:**
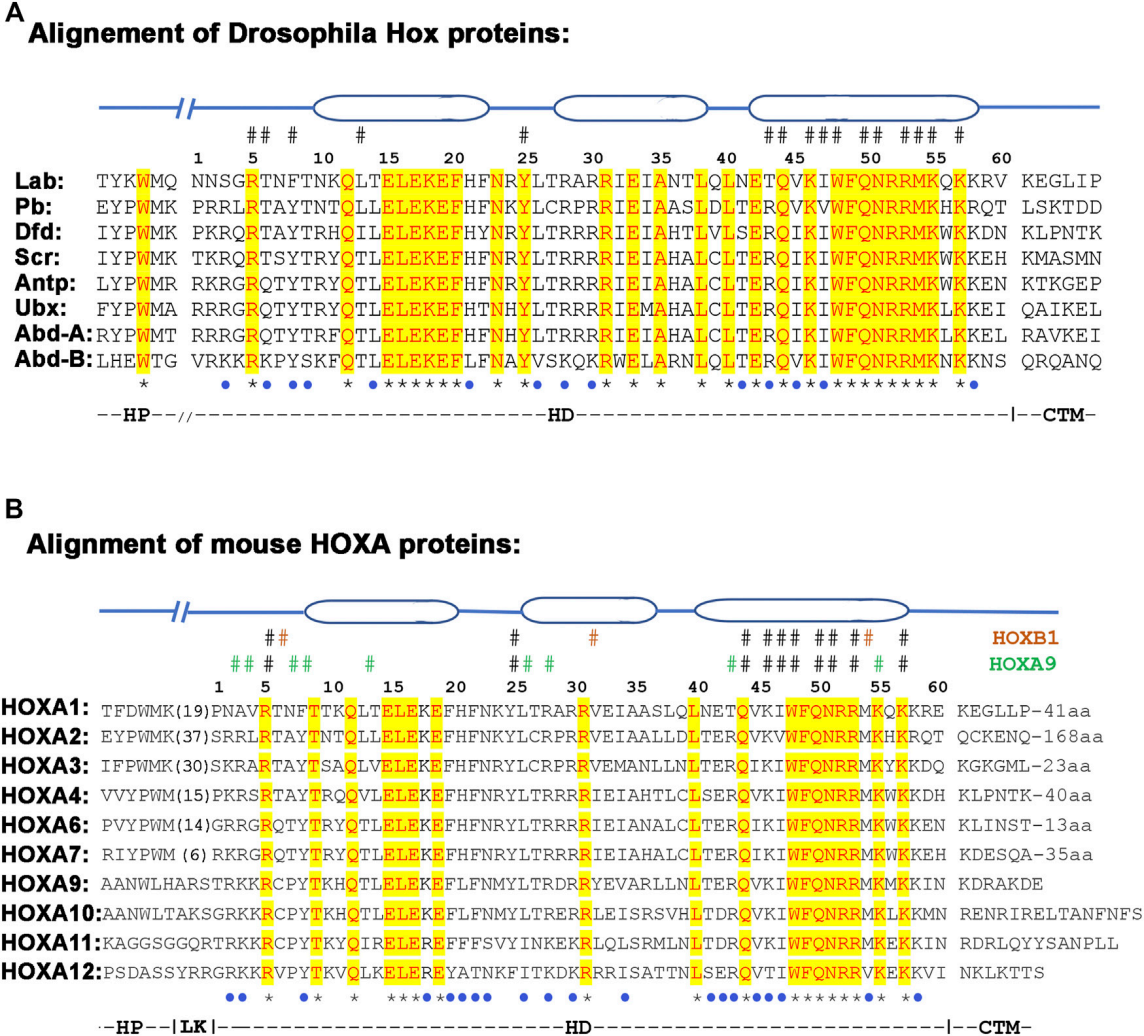
Alignment of hexapeptide (HP), linker (LK), homeodomain (HD) and CTM regions from Drosophila **(A)** and mouse **(B)** HOX proteins. The three ovel regions on the top of the alignment shows the position of alpha-helices. The ^#^ sign above the alignment depict amino acids that contact DNA, while star (*) and blue dot under the alignment show conservative and semi conservative amino acids respectively.

The HD stands out as the most conserved region of all HOX proteins (Figure 5). Three-dimensional structural studies using X-ray crystallography and NMR spectroscopy have revealed the presence of three alpha-helix regions in the HDs (Gehring et al., 1994b; Passner et al., 1999; Piper et al., 1999). The third helix, also known as the recognition helix, directly contacts the DNA through the major groove of DNA, while the region between the first and second helices establishes specific contact with the phosphate backbone. In addition, sequences adjacent to the N-terminus of the HD, referred to as the N-terminal extension region, also makes specific contact with DNA in the minor groove. These regions that insert into the minor groove have been shown to confer specificity to the HDs of *Drosophila* Ultrabithorax and Sex combs reduced Hox proteins imparting distinct DNA recognition properties (Joshi et al., 2007). The interactions of the N-terminal extension along with contacts mediated by the third helix are the primary determinates of DNA binding specificity and serve as a key constraint, maintaining the high level of sequence conservation.

Genetic and biochemical data suggest that the HD of HOX proteins is not freely interchangeable with each other. Functional assays of chimeric HOX proteins with swapped homeodomains of paralogs have been found to alter their functions, suggesting that homeodomains are not equivalent to each other (Zhao and Potter, 2001; Zhao and Potter, 2002). This indicates that the small differences between HD sequences contribute to differences in their DNA binding properties and functional activities (Furukubo-Tokunaga et al., 1993; Phelan et al., 1994; Zappavigna et al., 1994; Noyes et al., 2008; Breitinger et al., 2012). For example, 28 out of 60 amino acids are identical between all *Drosophila* HOX proteins and 18/60 are identical between all mouse HOXA paralogs (Figures 5A,B). In addition, many differences among the paralogs are conservative amino acid replacements that would be expected to preserve biochemical properties. This means that there are a series of non-conservative amino acid replacements or changes (19/60 in *Drosophila* and 22/60 in mouse HOXA paralogs), that have the potential to alter the DNA binding properties of HDs. For example, the crystal structures of HOXB1 and HOXA9 display many paralog specific amino acid contacts with DNA (Figure 5B) (Piper et al., 1999; LaRonde-LeBlanc and Wolberger, 2003). On the basis of sequence alignments and specific amino-acid residues within the homeodomain along with their relative positions in *Hox* clusters, *Hox* genes have been assigned to 14 different alloparalogous groups (PG1-14). These fall into three general classes: the anterior class contains PG1-5, the central class PG6-8 and the posterior class genes (PG9-14) (Domsch et al., 2015; Frobius and Funch, 2017). Hence, despite high conservation between homeodomain sequences there are paralog-specific differences that may alter DNA binding properties and preferences of HDs to modulate HOX function. The paralog specific changes in homeodomains may also alter the interaction of conserved amino acids with the DNA (Gruschus et al., 1997).

### Hexapeptide

The PBC group of proteins, *Drosophila* Extradenticle (Exd) and Homothorax (Hth) proteins and their vertebrate orthologs Pre-B-cell leukemia transcription factor (PBX1, PBX 2, PBX3 and PBX4) and myeloid ectopic leukemia virus integration site (MEIS1, MEIS2 and MEIS3) respectively, are well characterized cofactors of HOX proteins (Chan et al., 1994; Chang et al., 1995; Lu et al., 1995; Mann, 1995; Ryoo et al., 1999). Interactions with PBC proteins change DNA binding affinity and specificity of HOX monomer proteins (Slattery et al., 2011). *In vitro* binding analyses have demonstrated that the interaction between most HOX proteins and PBX are highly dependent on the HP motif, which contains the four core residues YPWM. This interaction alters the DNA binding properties of each protein, leading to the recognition of a bipartite HOX-PBC consensus site, and it enhances affinity and specificity of HOX target selection (van Dijk and Murre, 1994; Chang et al., 1995; Phelan et al., 1995; Popperl et al., 1995; Chan and Mann, 1996; Hudry et al., 2012).

The presence of the HP is important for HOX function, as loss of this motif has been implicated in the eliminating homeotic functions in several genes (*fushi-tarazu*, *zerknüllt*, *bicoid*) formed after duplication of *Hox* genes (Alonso et al., 2001; Panfilio and Akam, 2007). There is also evidence for sequence diversity among HP regions of different HOX proteins, which may impact the DNA binding properties and function of HOX proteins (Chang et al., 1995; Neuteboom et al., 1995; Medina-Martinez and Ramirez-Solis, 2003; Remacle et al., 2004). While the four core amino acids in the HP motif have been shown to be critical for its activity, only the tryptophan (W) residue in the 4^th^ position is highly conserved. The rest of the amino acids are highly variable among the HOX protein paralogs, which may alter their interactions with PBX (Figures 5A,B) (Neuteboom et al., 1995; Neuteboom and Murre, 1997; Mann et al., 2009). The divergence of the HP region away from YPWM motif in HOX proteins from paralogous groups 8–13 suggests a progressive change in their function through evolution of this motif (Figure 5B).

Consistent with the divergence of the HP regions*, in vitro* DNA binding experiments show that HOX proteins from paralog groups 1–10 mainly interact with PBX, while paralogy groups 11-13 preferentially interact with MEIS (Chan et al., 1994; Shen W. F. et al., 1997; Shen W.-F. et al., 1997). HOX proteins also form trimeric complex with PBX and MEIS proteins upon binding to target genes (Berthelsen et al., 1998; Shanmugam et al., 1999; Ferretti et al., 2000; Penkov et al., 2013; Amin et al., 2015). The presence of MEIS proteins have also been shown to induce remodeling of HOX-PBX interactions that leads to changes in the requirements for motifs that drive trimeric complex formation (Dard et al., 2019). High throughput SELEX-seq technology, which measures the relative affinities of transcription factor complexes with all possible DNA sequences, has shown that interaction of HOX proteins with Exd-Hth dimers alters the DNA binding properties of all eight *Drosophila* HOX proteins. Based on this data, the DNA binding specificities of HOX proteins can be subdivided into three main classes with similar DNA binding preferences: 1) anterior, containing Labial and Proboscipedia; 2) middle with Deformed and Sex comb reduced; and posterior, with Antennapedia, Ultrabithorax, Abdominal-A and Abdominal-B. These observations are consistent with the idea that the nature of interactions with PBX and MEIS varies among different HOX proteins and this directly impacts their DNA binding properties and functions.

Recent research has opened new insights into the complex nature of interactions between HOX and PBC proteins. Studies have revealed that novel HOX-PBX cofactor interactions arise through the loss or gain of other interacting domains beyond the HP and these have the potential to further modify HOX DNA binding properties and functions (Shen W. F. et al., 1997; Liu et al., 2008; Noro et al., 2011; Slattery et al., 2011; Hudry et al., 2012; Merabet and Mann, 2016; Dard et al., 2019; Singh et al., 2020). Use of sensitive *in vivo* assays to quantify HOX-PBX interactions have revealed that, in the presence of MEIS, the HP motif is dispensable in all HOX proteins except those from anterior paralog groups 1 and 2 (Dard et al., 2018). Furthermore, detailed analyses uncovered alternative PBC interaction motifs in human HOXB3, HOXA7 and HOXC8 proteins that are critical for HOX-PBC interaction in specific cell contexts and DNA-binding site topologies. While in the case of HOXA9, the HOXA9-PBX-MEIS interaction is dependent on the activity of the HP motif and two paralog-specific residues of the homeodomain region. These observations suggest that HOX-PBC interactions are not rigid and may behave in a dynamic manner that vary based on specific cellular and genomic contexts. The array of HOX- PBC interactions might have evolved independently in novel ways as a common regulatory node or mechanism to diversify DNA binding properties of HOX proteins. The highly conserved W residue in the HP region has been shown to be required for binding on HOX–PBX consensus motifs, while HOX protein binding on non-consensus motifs and low-affinity binding sites may be altered through HP diversity in combination with other novel interaction domains (Foos et al., 2015; Dard et al., 2018; Singh et al., 2020).

### Linker Region

HP region of HOX proteins is connected to the N-terminal of the HD through a linker (LK) region (Figures 5A,B). The sequence and size of the linker region is highly variable among the HOX paralogs, ranging from 3 to 50 amino acids in the vertebrate HOX proteins. Since the HP region of HOX proteins interacts with a highly conserved three amino acid loop extension domain in the HD region of PBX cofactors, the size and sequence of the linker region may constrain these interactions and further modulate DNA binding properties. Structural studies have revealed that the LK region of the *Drosophila* Sex Combs Reduced (Scr) HOX protein is critical for binding at some target sites, but on other binding targets it is disordered and makes minimal contribution to binding (Joshi et al., 2007). Converting the LK region of *Drosophila* Antp to that of Scr changes the DNA binding preference of the protein such that it binds very similar targets to those of Scr (Abe et al., 2015). Furthermore, comparative functional studies of mouse HOXA1 with HOXB1 proteins, Drosophila Ubx with Abd-A and Dfd with Scr show that the linker region is required for some aspects of the paralog specific functions of these HOX proteins (Merabet et al., 2003; Joshi et al., 2010; Singh et al., 2020). This indicates that the LK sequence has a role in modulating DNA binding preferences of HOX proteins and diversity in this region may be a determinant that underlies aspects of the paralog-specific functions of HOX proteins.

### C-Terminal Region

The C-terminal region flanking the homeodomain of HOX proteins is highly variable in size and sequence. The importance of this part of HOX proteins has been generally ignored because of the high degree of variability among the HOX paralogs. This region has also been left out of the studies that analyzed the three-dimensional structure of HOX proteins with PBX and DNA. Sequence analyses show that this region varies from 7 amino acids in HOXA13 to 168 amino acids in HOXA2 (Figure 5B). This is also highly variable between the HOXA alloparalogs (HOXA1 to HOXA13) and symparalogs among Hox A, B, C and D clusters. Our recent cross-species functional analyses revealed that a highly conserved CTM motif (KEGLLP) is a key determinant involved in maintaining the homologous ancestral functions of *Drosophila* Labial in the mouse HOXA1 protein (Figures 4A,C) (Singh et al., 2020). Diversification of this motif in mammalian homologs, HOXB1 and HOXD1led to a loss of the ancestral activity. Furthermore, structure prediction analyses suggested that the CTM region may establish another interaction domain with PBX1 on DNA. Consistent with this idea, *in vitro* DNA binding analyses revealed that the CTM region is not sufficient for HOX1 interaction with PBX1, but it can modulate the ability of HOXA1 to interact with PBX1 when bound to a target site (Singh et al., 2020). Similarly, small, conserved regions in the C-terminal domains of HOX proteins have also been observed in *Drosophila* HOX proteins Ubx and Abd-A (Lelli et al., 2011). *In vitro* DNA binding analyses and three-dimensional structures show that this region in Ubx (called Ubd-A region) is required for direct physical interaction with Exd, a homolog of PBX1, and affects DNA binding properties (Foos et al., 2015). Hence, it may play an analogous role to the CTM, identified in HOXA1. The UBD-A region is highly conserved among insect orthologs of Ubx, but it is absent from other arthropods and onychophorans (Galant and Carroll, 2002; Ronshaugen et al., 2002). Functional analyses of this domain with a transgenic reporter line displayed a repressive role on a Distal-less (*Dll*) *cis*-regulatory element that is involved in promoting limb development. These results suggests that evolution of the UBD-A domain suppressed limb formation in abdominal segments and provided an evolutionary transition to hexapod limb pattern. Furthermore, *in vitro* DNA binding and *in vivo* reporter assays show cooperation between linker and UBD-A region of the Ubx protein, which suggests subtle changes in HOX–PBC complexes have played a major role in the diversification of HOX protein function in evolution (Saadaoui et al., 2011). These data illustrate that flexible extensions outside of the HD helix have the potential to mediate additional contacts between HOX proteins and their cofactors in concert with those mediated by the HP motif on the opposing side of the DNA. Together these findings demonstrate that small differences in sequences outside of HDs, which do not contact DNA themselves, may be a common mechanism for modulating protein-protein interactions that impact DNA binding specificity of HOX proteins.

## Summary

A small number of changes in key amino acids may affect DNA binding properties and protein-protein interactions of transcription factors that can influence their DNA binding targets and potential for transcriptional activation or repression (Lai et al., 2001; Johnson et al., 2003; Chi, 2005; Sakazume et al., 2007; Shoubridge et al., 2012; Webb et al., 2012; Jubb et al., 2017; Singh et al., 2020; Chi, 2005). This indicates that sequence conservation alone may be a poor determinant in predicting the functions of HOX proteins. Genome-wide binding and gene expression analyses have revealed both overlapping and paralog-specific targets of HOX proteins (Hueber et al., 2007; Sorge et al., 2012; Beh et al., 2016; Bulajic et al., 2020; Singh et al., 2020). This suggests that there could be many common downstream targets in the genome, but the unique targets might have evolved by diversification of HOX proteins, resulting in selective alterations in their downstream target genes and inputs into novel gene regulatory programs. Paralog specific binding at unique target sites could arise through small differences in DNA binding domain and associated regions that alter interaction with cofactors such as PBX. Studies have shown that HOX-PBX interactions have diversified by altering interactions through the HP region and evolving novel contact points beyond it (Merabet et al., 2011; Saadaoui et al., 2011; Rivas et al., 2013). Altered HOX-PBX interactions may affect both DNA binding specificity of the HOX proteins and the transcriptional state of the target site. The diversification in the function of HOX proteins can be also introduced by changes outside the homeodomain and hexapeptide region (Chauvet et al., 2000; Gebelein et al., 2002; Singh et al., 2020). Several conserved short linear motifs (SLiMs) have been identified in HOX proteins that can often restrain the interaction potential of HOX proteins (Baeza et al., 2015). Deletion of SLiM motifs leads to loss, gain or interestingly enhanced interaction with cofactors that can alter regulatory potential of HOX proteins in a context specific manner. These dynamic changes in interaction with cofactors may alter Hox activity in tissue and cell type-specific manners which vary depending upon the cellular context (Capovilla et al., 1994; Joshi et al., 2010; Jung et al., 2014). These observations illustrate that a small number or subtle changes in multiple regions of HOX proteins can have a dramatic effect on their activity and may be an important feature that underlies the paralog specific functions by modifying DNA binding specificity and/or protein-protein interactions. Investigating the *in vivo* functional roles and evolution of other domains of HOX proteins beyond the HD should help to unravel how such similar proteins can exert diverse functions and be relevant in determining if this is a general mechanism used by other transcription factor families in the generation of diversity and evolution of novel functional activities of proteins.

## References

[B1] Abe N. Dror I. Yang L. Slattery M. Zhou T. Bussemaker H. J. (2015). Deconvolving the Recognition of DNA Shape from Sequence. Cell 161, 307–318. 10.1016/j.cell.2015.02.008 25843630 PMC4422406

[B2] Akam M. Averof M. Castelli-Gair J. Dawes R. Falciani F. Ferrier D. (1994). The Evolving Role of Hox Genes in Arthropods. Dev. Suppl. 1994, 209–215. 10.1242/dev.1994.supplement.209 7579521

[B3] Albalat R. Cañestro C. (2016). Evolution by Gene Loss. Nat. Rev. Genet. 17, 379–391. 10.1038/nrg.2016.39 27087500

[B4] Alonso C. R. Maxton-Kuechenmeister J. Akam M. (2001). Evolution of Ftz Protein Function in Insects. Curr. Biol. 11, 1473–1478. 10.1016/s0960-9822(01)00425-0 11566109

[B5] Amin S. Donaldson I. J. Zannino D. A. Hensman J. Rattray M. Losa M. (2015). Hoxa2 Selectively Enhances Meis Binding to Change a Branchial Arch Ground State. Develop. Cel 32, 265–277. 10.1016/j.devcel.2014.12.024 PMC4333904 25640223

[B6] Arendt D. (2018). Hox Genes and Body Segmentation. Science 361, 1310–1311. 10.1126/science.aav0692 30262483

[B7] Assaiya A. Burada A. P. Dhingra S. Kumar J. (2021). An Overview of the Recent Advances in Cryo-Electron Microscopy for Life Sciences. Emerg. Top. Life Sci. 5, 151–168. 10.1042/etls20200295 33760078

[B8] Averof M. Akam M. (1993). *HOM/Hox* Genes of *Artemia*: Implications for the Origin of Insect and Crustacean Body Plans. Curr. Biol. 3, 73–78. 10.1016/0960-9822(93)90158-k 15335797

[B9] Averof M. (2002). Arthropod Hox Genes: Insights on the Evolutionary Forces that Shape Gene Functions. Curr. Opin. Genet. Develop. 12, 386–392. 10.1016/s0959-437x(02)00314-3 12100881

[B10] Avsec Ž. Weilert M. Shrikumar A. Krueger S. Alexandari A. Dalal K. (2021). Base-resolution Models of Transcription-Factor Binding Reveal Soft Motif Syntax. Nat. Genet. 53, 354–366. 10.1038/s41588-021-00782-6 33603233 PMC8812996

[B11] Baëza M. Viala S. Heim M. Dard A. Hudry B. Duffraisse M. (2015). Inhibitory Activities of Short Linear Motifs Underlie Hox Interactome Specificity *In Vivo* . Elife 4, 6034. 10.7554/eLife.06034 PMC4392834 25869471

[B12] Banreti A. Hudry B. Sass M. Saurin A. J. Graba Y. (2014). Hox Proteins Mediate Developmental and Environmental Control of Autophagy. Develop. Cel 28, 56–69. 10.1016/j.devcel.2013.11.024 24389064

[B13] Beh C. Y. El-Sharnouby S. Chatzipli A. Russell S. Choo S. W. White R. (2016). Roles of Cofactors and Chromatin Accessibility in Hox Protein Target Specificity. Epigenetics. Chromatin 9, 1. 10.1186/s13072-015-0049-x 26753000 PMC4705621

[B14] Berthelsen J. Zappavigna V. Ferretti E. Mavilio F. Blasi F. (1998). The Novel Homeoprotein Prep1 Modulates Pbx-Hox Protein Cooperativity. Embo J. 17, 1434–1445. 10.1093/emboj/17.5.1434 9482740 PMC1170491

[B15] Biémont C. Vieira C. (2006). Junk DNA as an Evolutionary Force. Nature 443, 521–524. 10.1038/443521a 17024082

[B16] Blomme T. Vandepoele K. De Bodt S. Simillion C. Maere S. Van De Peer Y. (2006). The Gain and Loss of Genes during 600 Million Years of Vertebrate Evolution. Genome Biol. 7, R43. 10.1186/gb-2006-7-5-r43 16723033 PMC1779523

[B17] Braasch I. Bobe J. Guiguen Y. Postlethwait J. H. (2018). Reply to: 'Subfunctionalization versus Neofunctionalization after Whole-Genome Duplication'. Nat. Genet. 50, 910–911. 10.1038/s41588-018-0163-3 29955179

[B18] Breitinger C. Maethner E. Garcia-Cuellar M.-P. Slany R. K. (2012). The Homeodomain Region Controls the Phenotype of HOX-Induced Murine Leukemia. Blood 120, 4018–4027. 10.1182/blood-2011-10-384685 22990017

[B19] Bulajic M. Srivastava D. Dasen J. S. Wichterle H. Mahony S. Mazzoni E. O. (2020). Differential Abilities to Engage Inaccessible Chromatin Diversify Vertebrate Hox Binding Patterns. Development 147, dev194761. 10.1242/dev.194761 33028607 PMC7710020

[B20] Burki F. Kaessmann H. (2004). Birth and Adaptive Evolution of a Hominoid Gene that Supports High Neurotransmitter Flux. Nat. Genet. 36, 1061–1063. 10.1038/ng1431 15378063

[B21] Capovilla M. Brandt M. Botas J. (1994). Direct Regulation of Decapentaplegic by Ultrabithorax and its Role in Drosophila Midgut Morphogenesis. Cell 76, 461–475. 10.1016/0092-8674(94)90111-2 7906203

[B22] Carapuço M. Nóvoa A. Bobola N. Mallo M. (2005). Hox Genes Specify Vertebral Types in the Presomitic Mesoderm. Genes Dev. 19, 2116–2121. 10.1101/gad.338705 16166377 PMC1221883

[B23] Carroll S. B. (2008). Evo-devo and an Expanding Evolutionary Synthesis: a Genetic Theory of Morphological Evolution. Cell 134, 25–36. 10.1016/j.cell.2008.06.030 18614008

[B24] Carroll S. B. (1995). Homeotic Genes and the Evolution of Arthropods and Chordates. Nature 376, 479–485. 10.1038/376479a0 7637779

[B25] Chan S.-K. Jaffe L. Capovilla M. Botas J. Mann R. S. (1994). The DNA Binding Specificity of Ultrabithorax Is Modulated by Cooperative Interactions with Extradenticle, Another Homeoprotein. Cell 78, 603–615. 10.1016/0092-8674(94)90525-8 7915199

[B26] Chan S. K. Mann R. S. (1996). A Structural Model for a Homeotic Protein-Extradenticle-DNA Complex Accounts for the Choice of HOX Protein in the Heterodimer. Proc. Natl. Acad. Sci. U.S.A. 93, 5223–5228. 10.1073/pnas.93.11.5223 8643557 PMC39226

[B27] Chang C. P. Shen W. F. Rozenfeld S. Lawrence H. J. Largman C. Cleary M. L. (1995). Pbx Proteins Display Hexapeptide-dependent Cooperative DNA Binding with a Subset of Hox Proteins. Genes Dev. 9, 663–674. 10.1101/gad.9.6.663 7729685

[B28] Chauvet S. Merabet S. Bilder D. Scott M. P. Pradel J. Graba Y. (2000). Distinct Hox Protein Sequences Determine Specificity in Different Tissues. Proc. Natl. Acad. Sci. U.S.A. 97, 4064–4069. 10.1073/pnas.070046997 10737765 PMC18149

[B29] Chen F. Capecchi M. R. (1997). Targeted Mutations inHoxa-9andHoxb-9Reveal Synergistic Interactions. Develop. Biol. 181, 186–196. 10.1006/dbio.1996.8440 9013929

[B30] Chi Y.-I. (2005). Homeodomain Revisited: a Lesson from Disease-Causing Mutations. Hum. Genet. 116, 433–444. 10.1007/s00439-004-1252-1 15726414 PMC1579204

[B31] Clark A. G. (1994). Invasion and Maintenance of a Gene Duplication. Proc. Natl. Acad. Sci. U.S.A. 91, 2950–2954. 10.1073/pnas.91.8.2950 8159686 PMC43492

[B32] Coiffier D. Charroux B. Kerridge S. (2008). Common Functions of central and Posterior Hox Genes for the Repression of Head in the Trunk of Drosophila. Development 135, 291–300. 10.1242/dev.009662 18077590

[B33] Copley R. R. (2008). The Animal in the Genome: Comparative Genomics and Evolution. Phil. Trans. R. Soc. B 363, 1453–1461. 10.1098/rstb.2007.2235 18192189 PMC2614226

[B34] Dard A. Jia Y. Reboulet J. Bleicher F. Lavau C. Merabet S. (2019). The Human HOXA9 Protein Uses Paralog-specific Residues of the Homeodomain to Interact with TALE-Class Cofactors. Sci. Rep. 9, 5664. 10.1038/s41598-019-42096-y 30952900 PMC6450960

[B35] Dard A. Reboulet J. Jia Y. Bleicher F. Duffraisse M. Vanaker J.-M. (2018). Human HOX Proteins Use Diverse and Context-dependent Motifs to Interact with TALE Class Cofactors. Cel Rep. 22, 3058–3071. 10.1016/j.celrep.2018.02.070 29539431

[B36] Davenne M. Maconochie M. K. Neun R. Pattyn A. Chambon P. Krumlauf R. (1999). *Hoxa2* and *Hoxb2* Control Dorsoventral Patterns of Neuronal Development in the Rostral Hindbrain. Neuron 22, 677–691. 10.1016/s0896-6273(00)80728-x 10230789

[B37] Debodt S. Maere S. Vandepeer Y. (2005). Genome Duplication and the Origin of Angiosperms. Trends Ecol. Evol. 20, 591–597. 10.1016/j.tree.2005.07.008 16701441

[B38] Dehal P. Boore J. L. (2005). Two Rounds of Whole Genome Duplication in the Ancestral Vertebrate. Plos Biol. 3, e314. 10.1371/journal.pbio.0030314 16128622 PMC1197285

[B39] Denans N. Iimura T. Pourquié O. (2015). Hox Genes Control Vertebrate Body Elongation by Collinear Wnt Repression. Elife 4. 10.7554/eLife.04379 PMC4384752 25719209

[B40] Desplan C. Theis J. O'farrell P. H. (1988). The Sequence Specificity of Homeodomain-DNA Interaction. Cell 54, 1081–1090. 10.1016/0092-8674(88)90123-7 3046753 PMC2753412

[B41] Domsch K. Papagiannouli F. Lohmann I. (2015). The HOX-Apoptosis Regulatory Interplay in Development and Disease. Curr. Top. Dev. Biol. 114, 121–158. 10.1016/bs.ctdb.2015.07.014 26431566

[B42] Doudna J. A. Charpentier E. (2014). The New Frontier of Genome Engineering with CRISPR-Cas9. Science 346, 1258096. 10.1126/science.1258096 25430774

[B43] Duboule D. Dollé P. (1989). The Structural and Functional Organization of the Murine *HOX* Gene Family Resembles that of *Drosophila* Homeotic Genes. EMBO J. 8, 1497–1505. 10.1002/j.1460-2075.1989.tb03534.x 2569969 PMC400980

[B44] Duboule D. (2007). The Rise and Fall of Hox Gene Clusters. Development 134, 2549–2560. 10.1242/dev.001065 17553908

[B45] Duboule D. (1998). Vertebrate Hox Gene Regulation: Clustering And/or Colinearity? Curr. Opin. Genet. Develop. 8, 514–518. 10.1016/s0959-437x(98)80004-x 9794816

[B46] Enard W. Gehre S. Hammerschmidt K. Hölter S. M. Blass T. Somel M. (2009). A Humanized Version of Foxp2 Affects Cortico-Basal Ganglia Circuits in Mice. Cell 137, 961–971. 10.1016/j.cell.2009.03.041 19490899

[B47] Enriquez J. Boukhatmi H. Dubois L. Philippakis A. A. Bulyk M. L. Michelson A. M. (2010). Multi-step Control of Muscle Diversity by Hox Proteins in the Drosophila Embryo. Development 137, 457–466. 10.1242/dev.045286 20056681 PMC2858909

[B48] Escriva H. Bertrand S. Germain P. Robinson-Rechavi M. Umbhauer M. Cartry J. (2006). Neofunctionalization in Vertebrates: the Example of Retinoic Acid Receptors. Plos Genet. 2, e102. 10.1371/journal.pgen.0020102 16839186 PMC1500811

[B49] Fernández R. Gabaldón T. (2020). Gene Gain and Loss across the Metazoan Tree of Life. Nat. Ecol. Evol. 4, 524–533. 10.1038/s41559-019-1069-x 31988444 PMC7124887

[B50] Ferretti E. Marshall H. Pöpperl H. Maconochie M. Krumlauf R. Blasi F. (2000). Segmental Expression of Hoxb2 in R4 Requires Two Separate Sites that Integrate Cooperative Interactions between Prep1, Pbx and Hox Proteins. Development 127, 155–166. 10.1242/dev.127.1.155 10654609

[B51] Foos N. Maurel-Zaffran C. Maté M. J. Vincentelli R. Hainaut M. Berenger H. (2015). A Flexible Extension of the Drosophila Ultrabithorax Homeodomain Defines a Novel Hox/PBC Interaction Mode. Structure 23, 270–279. 10.1016/j.str.2014.12.011 25651060

[B52] Force A. Lynch M. Pickett F. B. Amores A. Yan Y.-l. Postlethwait J. (1999). Preservation of Duplicate Genes by Complementary, Degenerative Mutations. Genetics 151, 1531–1545. 10.1093/genetics/151.4.1531 10101175 PMC1460548

[B53] Franchini L. F. Pollard K. S. (2017). Human Evolution: the Non-coding Revolution. BMC Biol. 15, 89. 10.1186/s12915-017-0428-9 28969617 PMC5625771

[B54] Friedman R. Hughes A. L. (2001). Pattern and Timing of Gene Duplication in Animal Genomes. Genome Res. 11, 1842–1847. 10.1101/gr.200601 11691848 PMC311158

[B55] Fröbius A. C. Funch P. (2017). Rotiferan Hox Genes Give New Insights into the Evolution of Metazoan Bodyplans. Nat. Commun. 8, 9. 10.1038/s41467-017-00020-w 28377584 PMC5431905

[B56] Fromental-Ramain C. Warot X. Lakkaraju S. Favier B. Haack H. Birling C. (1996). Specific and Redundant Functions of the Paralogous Hoxa-9 and Hoxd-9 Genes in Forelimb and Axial Skeleton Patterning. Development 122, 461–472. 10.1242/dev.122.2.461 8625797

[B57] Furukubo-Tokunaga K. Flister S. Gehring W. J. (1993). Functional Specificity of the Antennapedia Homeodomain. Proc. Natl. Acad. Sci. U.S.A. 90, 6360–6364. 10.1073/pnas.90.13.6360 8101003 PMC46928

[B58] Galant R. Carroll S. B. (2002). Evolution of a Transcriptional Repression Domain in an Insect Hox Protein. Nature 415, 910–913. 10.1038/nature717 11859369

[B59] Galant R. Walsh C. M. Carroll S. B. (2002). Hox Repression of a Target Gene: Extradenticle-independent, Additive Action through Multiple Monomer Binding Sites. Development 129, 3115–3126. 10.1242/dev.129.13.3115 12070087

[B60] Garcia-Bellido A. Ripoll P. Morata G. (1973). Developmental Compartmentalisation of the wing Disk of Drosophila. Nat. New Biol. 245, 251–253. 10.1038/newbio245251a0 4518369

[B61] Garcia-Fernàndez J. Holland P. W. H. (1994). Archetypal Organization of the Amphioxus Hox Gene Cluster. Nature 370, 563–566. 10.1038/370563a0 7914353

[B62] Gasperskaja E. Kučinskas V. (2017). The Most Common Technologies and Tools for Functional Genome Analysis. Aml 24, 1–11. 10.6001/actamedica.v24i1.3457 PMC5467957 28630587

[B63] Gebelein B. Culi J. Ryoo H. D. Zhang W. Mann R. S. (2002). Specificity of Distalless Repression and Limb Primordia Development by Abdominal Hox Proteins. Develop. Cel 3, 487–498. 10.1016/s1534-5807(02)00257-5 12408801

[B64] Gebelein B. Mckay D. J. Mann R. S. (2004). Direct Integration of Hox and Segmentation Gene Inputs during Drosophila Development. Nature 431, 653–659. 10.1038/nature02946 15470419

[B65] Gehring W. J. Affolter M. Bürglin T. (1994a). Homeodomain Proteins. Annu. Rev. Biochem. 63, 487–526. 10.1146/annurev.bi.63.070194.002415 7979246

[B66] Gehring W. J. Qian Y. Q. Billeter M. Furukubo-Tokunaga K. Schier A. F. Resendez-Perez D. (1994b). Homeodomain-DNA Recognition. Cell 78, 211–223. 10.1016/0092-8674(94)90292-5 8044836

[B67] Graham A. Papalopulu N. Krumlauf R. (1989). The Murine and *Drosophila* Homeobox Gene Complexes Have Common Features of Organization and Expression. Cell 57, 367–378. 10.1016/0092-8674(89)90912-4 2566383

[B68] Greer J. M. Puetz J. Thomas K. R. Capecchi M. R. (2000). Maintenance of Functional Equivalence during Paralogous Hox Gene Evolution. Nature 403, 661–665. 10.1038/35001077 10688203

[B69] Gruschus J. M. Tsao D. H. H. Wang L.-H. Nirenberg M. Ferretti J. A. (1997). Interactions of the vnd/NK-2 Homeodomain with DNA by Nuclear Magnetic Resonance Spectroscopy: Basis of Binding Specificity. Biochemistry 36, 5372–5380. 10.1021/bi9620060 9154919

[B70] Guijarro-Clarke C. Holland P. W. H. Paps J. (2020a). Publisher Correction: Widespread Patterns of Gene Loss in the Evolution of the Animal Kingdom. Nat. Ecol. Evol. 4, 661. 10.1038/s41559-020-1159-9 32108759

[B71] Guijarro-Clarke C. Holland P. W. H. Paps J. (2020b). Widespread Patterns of Gene Loss in the Evolution of the Animal Kingdom. Nat. Ecol. Evol. 4, 519–523. 10.1038/s41559-020-1129-2 32094540

[B72] Guo T. Mandai K. Condie B. G. Wickramasinghe S. R. Capecchi M. R. Ginty D. D. (2011). An Evolving NGF-Hoxd1 Signaling Pathway Mediates Development of Divergent Neural Circuits in Vertebrates. Nat. Neurosci. 14, 31–36. 10.1038/nn.2710 21151121 PMC3180918

[B73] Hahn M. W. Wray G. A. (2002). The G-Value Paradox. Evol. Dev. 4, 73–75. 10.1046/j.1525-142x.2002.01069.x 12004964

[B74] Hanks M. C. Loomis C. A. Harris E. Tong C. X. Anson-Cartwright L. Auerbach A. (1998). Drosophila Engrailed Can Substitute for Mouse Engrailed1 Function in Mid-hindbrain, but Not Limb Development. Development 125, 4521–4530. 10.1242/dev.125.22.4521 9778510

[B75] He Q. Johnston J. Zeitlinger J. (2015). ChIP-nexus Enables Improved Detection of *In Vivo* Transcription Factor Binding Footprints. Nat. Biotechnol. 33, 395–401. 10.1038/nbt.3121 25751057 PMC4390430

[B76] He S. Del Viso F. Chen C.-Y. Ikmi A. Kroesen A. E. Gibson M. C. (2018). An Axial Hox Code Controls Tissue Segmentation and Body Patterning in *Nematostella vectensis* . Science 361, 1377–1380. 10.1126/science.aar8384 30262503

[B77] He X. Zhang J. (2005). Rapid Subfunctionalization Accompanied by Prolonged and Substantial Neofunctionalization in Duplicate Gene Evolution. Genetics 169, 1157–1164. 10.1534/genetics.104.037051 15654095 PMC1449125

[B78] Hedges S. B. Marin J. Suleski M. Paymer M. Kumar S. (2015). Tree of Life Reveals Clock-like Speciation and Diversification. Mol. Biol. Evol. 32, 835–845. 10.1093/molbev/msv037 25739733 PMC4379413

[B79] Hirth F. Loop T. Egger B. Miller D. F. B. Kaufman T. C. Reichert H. (2001). Functional Equivalence of Hox Gene Products in the Specification of the Tritocerebrum during Embryonic Brain Development of Drosophila. Development 128, 4781–4788. 10.1242/dev.128.23.4781 11731458

[B80] Hoegg S. Meyer A. (2005). Hox Clusters as Models for Vertebrate Genome Evolution. Trends Genet. 21, 421–424. 10.1016/j.tig.2005.06.004 15967537

[B81] Holland L. Z. Ocampo Daza D. (2018). A New Look at an Old Question: when Did the Second Whole Genome Duplication Occur in Vertebrate Evolution? Genome Biol. 19, 209. 10.1186/s13059-018-1592-0 30486862 PMC6260733

[B82] Holland P. W. H. Garcia-Fernàndez J. Williams N. A. Sidow A. (1994). Gene Duplications and the Origins of Vertebrate Development. Dev. Suppl. 1994, 125–133. 10.1242/dev.1994.supplement.125 7579513

[B83] Horan G. S. Ramírez-Solis R. Featherstone M. S. Wolgemuth D. J. Bradley A. Behringer R. R. (1995). Compound Mutants for the Paralogous *Hoxa-4, Hoxb-4*, and *Hoxd-4* Genes Show More Complete Homeotic Transformations and a Dose-dependent Increase in the Number of Vertebrae Transformed. Genes Dev. 9, 1667–1677. 10.1101/gad.9.13.1667 7628700

[B84] Hudry B. Remacle S. Delfini M.-C. Rezsohazy R. Graba Y. Merabet S. (2012). Hox Proteins Display a Common and Ancestral Ability to Diversify Their Interaction Mode with the PBC Class Cofactors. Plos Biol. 10, e1001351. 10.1371/journal.pbio.1001351 22745600 PMC3383740

[B85] Hueber S. D. Bezdan D. Henz S. R. Blank M. Wu H. Lohmann I. (2007). Comparative Analysis of Hox Downstream Genes inDrosophila. Development 134, 381–392. 10.1242/dev.02746 17166915

[B86] Hughes A. L. (1994). The Evolution of Functionally Novel Proteins after Gene Duplication. Proc. Biol. Sci. 256, 119–124. 10.1098/rspb.1994.0058 8029240

[B87] Hunter M. P. Prince V. E. (2002). Zebrafish Hox Paralogue Group 2 Genes Function Redundantly as Selector Genes to Pattern the Second Pharyngeal Arch. Develop. Biol. 247, 367–389. 10.1006/dbio.2002.0701 12086473

[B88] Iacovino M. Hernandez C. Xu Z. Bajwa G. Prather M. Kyba M. (2009). A Conserved Role for Hox Paralog Group 4 in Regulation of Hematopoietic Progenitors. Stem Cell Develop. 18, 783–792. 10.1089/scd.2008.0227 PMC2775089 18808325

[B89] Ikuta T. (2011). Evolution of Invertebrate Deuterostomes and Hox/ParaHox Genes. Genomics. Proteomics. Bioinformatics 9, 77–96. 10.1016/s1672-0229(11)60011-9 21802045 PMC5054439

[B90] Innan H. (2009). Population Genetic Models of Duplicated Genes. Genetica 137, 19–37. 10.1007/s10709-009-9355-1 19266289

[B91] Irie N. Satoh N. Kuratani S. (2018). The Phylum Vertebrata: a Case for Zoological Recognition. Zoolog. Lett 4, 32. 10.1186/s40851-018-0114-y PMC6307173 30607258

[B92] Johnson D. Kan S.-h. Oldridge M. Trembath R. C. Roche P. Esnouf R. M. (2003). Missense Mutations in the Homeodomain of HOXD13 Are Associated with Brachydactyly Types D and E. Am. J. Hum. Genet. 72, 984–997. 10.1086/374721 12649808 PMC1180360

[B93] Joshi R. Passner J. M. Rohs R. Jain R. Sosinsky A. Crickmore M. A. (2007). Functional Specificity of a Hox Protein Mediated by the Recognition of Minor Groove Structure. Cell 131, 530–543. 10.1016/j.cell.2007.09.024 17981120 PMC2709780

[B94] Joshi R. Sun L. Mann R. (2010). Dissecting the Functional Specificities of Two Hox Proteins. Genes Dev. 24, 1533–1545. 10.1101/gad.1936910 20634319 PMC2904943

[B95] Jubb H. C. Pandurangan A. P. Turner M. A. Ochoa-Montaño B. Blundell T. L. Ascher D. B. (2017). Mutations at Protein-Protein Interfaces: Small Changes over Big Surfaces Have Large Impacts on Human Health. Prog. Biophys. Mol. Biol. 128, 3–13. 10.1016/j.pbiomolbio.2016.10.002 27913149

[B96] Jumper J. Evans R. Pritzel A. Green T. Figurnov M. Ronneberger O. (2021). Highly Accurate Protein Structure Prediction with AlphaFold. Nature 596, 583–589. 10.1038/s41586-021-03819-2 34265844 PMC8371605

[B97] Jung H. Mazzoni E. O. Soshnikova N. Hanley O. Venkatesh B. Duboule D. (2014). Evolving Hox Activity Profiles Govern Diversity in Locomotor Systems. Develop. Cel 29, 171–187. 10.1016/j.devcel.2014.03.008 PMC4024207 24746670

[B98] Kaufman T. C. Lewis R. Wakimoto B. (1980). Cytogenetic Analysis of Chromosome 3 in Drosophila Melanogaster: The Homoeotic Gene Complex in Polytene Chromosome Interval 84a-B. Genetics 94, 115–133. 10.1093/genetics/94.1.115 17248988 PMC1214128

[B99] Kleinjan D. A. Bancewicz R. M. Gautier P. Dahm R. Schonthaler H. B. Damante G. (2008). Subfunctionalization of Duplicated Zebrafish Pax6 Genes by Cis-Regulatory Divergence. Plos Genet. 4, e29. 10.1371/journal.pgen.0040029 18282108 PMC2242813

[B100] Kmita M. Duboule D. (2003). Organizing Axes in Time and Space; 25 Years of Colinear Tinkering. Science 301, 331–333. 10.1126/science.1085753 12869751

[B101] Knoepfler P. S. Kamps M. P. (1995). The Pentapeptide Motif of Hox Proteins Is Required for Cooperative DNA Binding with Pbx1, Physically Contacts Pbx1, and Enhances DNA Binding by Pbx1. Mol. Cel Biol 15, 5811–5819. 10.1128/mcb.15.10.5811 PMC230833 7565734

[B102] Koonin E. V. (2005). Orthologs, Paralogs, and Evolutionary Genomics. Annu. Rev. Genet. 39, 309–338. 10.1146/annurev.genet.39.073003.114725 16285863

[B103] Krumlauf R. (1994). *Hox* Genes in Vertebrate Development. Cell 78, 191–201. 10.1016/0092-8674(94)90290-9 7913880

[B104] Kuraku S. Meyer A. (2009). The Evolution and Maintenance of Hox Gene Clusters in Vertebrates and the Teleost-specific Genome Duplication. Int. J. Dev. Biol. 53, 765–773. 10.1387/ijdb.072533km 19557682

[B105] Lacombe J. Hanley O. Jung H. Philippidou P. Surmeli G. Grinstein J. (2013). Genetic and Functional Modularity of Hox Activities in the Specification of Limb-Innervating Motor Neurons. Plos Genet. 9, e1003184. 10.1371/journal.pgen.1003184 23359544 PMC3554521

[B106] Lai C. S. L. Fisher S. E. Hurst J. A. Vargha-Khadem F. Monaco A. P. (2001). A Forkhead-Domain Gene Is Mutated in a Severe Speech and Language Disorder. Nature 413, 519–523. 10.1038/35097076 11586359

[B107] Laity J. H. Lee B. M. Wright P. E. (2001). Zinc finger Proteins: New Insights into Structural and Functional Diversity. Curr. Opin. Struct. Biol. 11, 39–46. 10.1016/s0959-440x(00)00167-6 11179890

[B108] Lamka M. L. Boulet A. M. Sakonju S. (1992). Ectopic Expression of UBX and ABD-B Proteins during Drosophila Embryogenesis: Competition, Not a Functional Hierarchy, Explains Phenotypic Suppression. Development 116, 841–854. 10.1242/dev.116.4.841 1363544

[B109] Laronde-Leblanc N. A. Wolberger C. (2003). Structure of HoxA9 and Pbx1 Bound to DNA: Hox Hexapeptide and DNA Recognition Anterior to Posterior. Genes Dev. 17, 2060–2072. 10.1101/gad.1103303 12923056 PMC196259

[B110] Laurent J. M. Garge R. K. Teufel A. I. Wilke C. O. Kachroo A. H. Marcotte E. M. (2020). Humanization of Yeast Genes with Multiple Human Orthologs Reveals Functional Divergence between Paralogs. Plos Biol. 18, e3000627. 10.1371/journal.pbio.3000627 32421706 PMC7259792

[B111] Lawrence P. A. Morata G. (1994). Homeobox Genes: Their Function in Drosophila Segmentation and Pattern Formation. Cell 78, 181–189. 10.1016/0092-8674(94)90289-5 7913879

[B112] Lelli K. M. Noro B. Mann R. S. (2011). Variable Motif Utilization in Homeotic Selector (Hox)-Cofactor Complex Formation Controls Specificity. Proc. Natl. Acad. Sci. U.S.A. 108, 21122–21127. 10.1073/pnas.1114118109 22160705 PMC3248519

[B113] Lemons D. Mcginnis W. (2006). Genomic Evolution of Hox Gene Clusters. Science 313, 1918–1922. 10.1126/science.1132040 17008523

[B114] Lewis E. B. (1978). A Gene Complex Controlling Segmentation in Drosophila. Nature 276, 565–570. 10.1038/276565a0 103000

[B115] Lewis E. B. (1994). Homeosis: the First 100 Years. Trends Genet. 10, 341–343. 10.1016/0168-9525(94)90117-1 7985234

[B116] Li X. Mcginnis W. (1999). Activity Regulation of Hox Proteins, a Mechanism for Altering Functional Specificity in Development and Evolution. Proc. Natl. Acad. Sci. U.S.A. 96, 6802–6807. 10.1073/pnas.96.12.6802 10359793 PMC21996

[B117] Li X. Murre C. Mcginnis W. (1999). Activity Regulation of a Hox Protein and a Role for the Homeodomain in Inhibiting Transcriptional Activation. Embo J. 18, 198–211. 10.1093/emboj/18.1.198 9878063 PMC1171115

[B118] Liu Y. Matthews K. S. Bondos S. E. (2008). Multiple Intrinsically Disordered Sequences Alter DNA Binding by the Homeodomain of the Drosophila Hox Protein Ultrabithorax. J. Biol. Chem. 283, 20874–20887. 10.1074/jbc.m800375200 18508761 PMC2475714

[B119] Loker R. Sanner J. E. Mann R. S. (2021). Cell-type-specific Hox Regulatory Strategies Orchestrate Tissue Identity. Curr. Biol. 10.1016/j.cub.2021.07.030 PMC8511240 34358443

[B120] Lu Q. Knoepfler P. Scheele J. Wright D. Kamps M. (1995). Both Pbx1 and E2A-Pbx1 Bind the DNA Motif ACCAATCAA Cooperatively with the Products of Mutliple Murine HOX Genes, Some of Which Themselves Are Oncogenes. Mol. Cel Biol 15, 3786. 10.1128/mcb.15.7.3786 PMC230617 7791786

[B121] Lundin L. G. (1999). Gene Duplications in Early Metazoan Evolution. Semin. Cel Develop. Biol. 10, 523–530. 10.1006/scdb.1999.0333 10597636

[B122] Lutz B. Lu H. C. Eichele G. Miller D. Kaufman T. C. (1996). Rescue of *Drosophila Labial* Null Mutant by the Chicken Ortholog *Hoxb-1* Demonstrates that the Function of *Hox* Genes Is Phylogenetically Conserved. Genes Dev. 10, 176–184. 10.1101/gad.10.2.176 8566751

[B123] Lynch M. Conery J. S. (2000). The Evolutionary Fate and Consequences of Duplicate Genes. Science 290, 1151–1155. 10.1126/science.290.5494.1151 11073452

[B124] Lynch M. Force A. (2000). The Probability of Duplicate Gene Preservation by Subfunctionalization. Genetics 154, 459–473. 10.1093/genetics/154.1.459 10629003 PMC1460895

[B125] Maconochie M. Nonchev S. Morrison A. Krumlauf R. (1996). Paralogous *Hox* Genes: Function and Regulation. Annu. Rev. Genet. 30, 529–556. 10.1146/annurev.genet.30.1.529 8982464

[B126] Maeda R. K. Karch F. (2006). The ABC of the BX-C: the Bithorax Complex Explained. Development 133, 1413–1422. 10.1242/dev.02323 16556913

[B127] Malicki J. Schughart K. Mcginnis W. (1990). Mouse Hox-2.2 Specifies Thoracic Segmental Identity in Drosophila Embryos and Larvae. Cell 63, 961–967. 10.1016/0092-8674(90)90499-5 1979525

[B128] Manley N. R. Capecchi M. R. (1997). *Hox* Group 3 Paralogous Genes Act Synergistically in the Formation of Somitic and Neural Crest-Derived Structures. Develop. Biol. 192, 274–288. 10.1006/dbio.1997.8765 9441667

[B129] Mann R. S. Chan S.-K. (1996). Extra Specificity from Extradenticle: the Partnership between HOX and PBX/EXD Homeodomain Proteins. Trends Genet. 12, 258–262. 10.1016/0168-9525(96)10026-3 8763497

[B130] Mann R. S. Lelli K. M. Joshi R. (2009). Chapter 3 Hox Specificity. Curr. Top. Dev. Biol. 88, 63–101. 10.1016/s0070-2153(09)88003-4 19651302 PMC2810641

[B131] Mann R. S. (1995). The Specificity of Homeotic Gene Function. Bioessays 17, 855–863. 10.1002/bies.950171007 7487967

[B132] Marcussen T. Oxelman B. Skog A. Jakobsen K. S. (2010). Evolution of Plant RNA Polymerase IV/V Genes: Evidence of Subneofunctionalization of Duplicated NRPD2/NRPE2-like Paralogs in Viola (Violaceae). BMC Evol. Biol. 10, 45. 10.1186/1471-2148-10-45 20158916 PMC2834690

[B133] Mazet F. M. Shimeld S. (2002). Gene Duplication and Divergence in the Early Evolution of Vertebrates. Curr. Opin. Genet. Develop. 12, 393–396. 10.1016/s0959-437x(02)00315-5 12100882

[B134] Mcginnis N. Kuziora M. A. Mcginnis W. (1990). Human Hox-4.2 and Drosophila Deformed Encode Similar Regulatory Specificities in Drosophila Embryos and Larvae. Cell 63, 969–976. 10.1016/0092-8674(90)90500-e 1979526

[B135] Mcginnis W. Krumlauf R. (1992). Homeobox Genes and Axial Patterning. Cell 68, 283–302. 10.1016/0092-8674(92)90471-n 1346368

[B136] Mclain K. Schreiner C. Yager K. L. Stock J. L. Steven Potter S. (1992). Ectopic Expression of Hox-2.3 Induces Craniofacial and Skeletal Malformations in Transgenic Mice. Mech. Develop. 39, 3–16. 10.1016/0925-4773(92)90021-b 1362649

[B137] Mcnulty C. L. Peres J. N. Bardine N. Van Den Akker W. M. R. Durston A. J. (2005). Knockdown of the Complete Hox Paralogous Group 1 Leads to Dramatic Hindbrain and Neural Crest Defects. Development 132, 2861–2871. 10.1242/dev.01872 15930115

[B138] Medina-Martinez O. Bradley A. Ramírez-Solis R. (2000). A Large Targeted Deletion of Hoxb1-Hoxb9 Produces a Series Single-Segment Anterior Homeotic Transformations. Develop. Biol. 222, 71–83. 10.1006/dbio.2000.9683 10885747

[B139] Medina-Martínez O. Ramírez-Solis R. (2003). *In Vivo* mutagenesis of the Hoxb8 Hexapeptide Domain Leads to Dominant Homeotic Transformations that Mimic the Loss-Of-Function Mutations in Genes of the Hoxb Cluster. Develop. Biol. 264, 77–90. 10.1016/j.ydbio.2003.07.020 14623233

[B140] Merabet S. Kambris Z. Capovilla M. Bérenger H. Pradel J. Graba Y. (2003). The Hexapeptide and Linker Regions of the AbdA Hox Protein Regulate its Activating and Repressive Functions. Develop. Cel 4, 761–768. 10.1016/s1534-5807(03)00126-6 12737810

[B141] Merabet S. Litim-Mecheri I. Karlsson D. Dixit R. Saadaoui M. Monier B. (2011). Insights into Hox Protein Function from a Large Scale Combinatorial Analysis of Protein Domains. Plos Genet. 7, e1002302. 10.1371/journal.pgen.1002302 22046139 PMC3203194

[B142] Merabet S. Mann R. S. (2016). To Be Specific or Not: The Critical Relationship between Hox and TALE Proteins. Trends Genet. 32, 334–347. 10.1016/j.tig.2016.03.004 27066866 PMC4875764

[B143] Miguez A. Ducret S. Di Meglio T. Parras C. Hmidan H. Haton C. (2012). Opposing Roles for Hoxa2 and Hoxb2 in Hindbrain Oligodendrocyte Patterning. J. Neurosci. 32, 17172–17185. 10.1523/jneurosci.0885-12.2012 23197710 PMC6621859

[B144] Mora C. Tittensor D. P. Adl S. Simpson A. G. B. Worm B. (2011). How many Species Are There on Earth and in the Ocean? Plos Biol. 9, e1001127. 10.1371/journal.pbio.1001127 21886479 PMC3160336

[B145] Nadeau J. H. Sankoff D. (1997). Comparable Rates of Gene Loss and Functional Divergence after Genome Duplications Early in Vertebrate Evolution. Genetics 147, 1259–1266. 10.1093/genetics/147.3.1259 9383068 PMC1208249

[B146] Neuteboom S. T. Murre C. (1997). Pbx Raises the DNA Binding Specificity but Not the Selectivity of Antennapedia Hox Proteins. Mol. Cel Biol 17, 4696–4706. 10.1128/mcb.17.8.4696 PMC232322 9234726

[B147] Neuteboom S. T. Peltenburg L. T. Van Dijk M. A. Murre C. (1995). The Hexapeptide LFPWMR in Hoxb-8 Is Required for Cooperative DNA Binding with Pbx1 and Pbx2 Proteins. Proc. Natl. Acad. Sci. U.S.A. 92, 9166–9170. 10.1073/pnas.92.20.9166 7568094 PMC40945

[B148] Noro B. Lelli K. Sun L. Mann R. S. (2011). Competition for Cofactor-dependent DNA Binding Underlies Hox Phenotypic Suppression. Genes Dev. 25, 2327–2332. 10.1101/gad.175539.111 22085961 PMC3222899

[B149] Noyes M. B. Christensen R. G. Wakabayashi A. Stormo G. D. Brodsky M. H. Wolfe S. A. (2008). Analysis of Homeodomain Specificities Allows the Family-wide Prediction of Preferred Recognition Sites. Cell 133, 1277–1289. 10.1016/j.cell.2008.05.023 18585360 PMC2478728

[B150] Ohno S. (1970). Evolution by Gene Duplication. Springer-Verlag Berlin Heidelberg, 160.

[B151] Panfilio K. A. Akam M. (2007). A Comparison of Hox3 and Zen Protein Coding Sequences in Taxa that Span the Hox3/zen Divergence. Dev. Genes Evol. 217, 323–329. 10.1007/s00427-007-0133-8 17285343

[B152] Panopoulou G. Hennig S. Groth D. Krause A. Poustka A. J. Herwig R. (2003). New Evidence for Genome-wide Duplications at the Origin of Vertebrates Using an Amphioxus Gene Set and Completed Animal Genomes. Genome Res. 13, 1056–1066. 10.1101/gr.874803 12799346 PMC403660

[B153] Paps J. Holland P. W. H. (2018). Reconstruction of the Ancestral Metazoan Genome Reveals an Increase in Genomic novelty. Nat. Commun. 9, 1730. 10.1038/s41467-018-04136-5 29712911 PMC5928047

[B154] Pascual-Anaya J. D’Aniello S. Kuratani S. Garcia-Fernàndez J. (2013). Evolution of Hox Gene Clusters in Deuterostomes. BMC Develop. Biol. 13, 26. 10.1186/1471-213x-13-26 PMC3707753 23819519

[B155] Passner J. M. Ryoo H. D. Shen L. Mann R. S. Aggarwal A. K. (1999). Structure of a DNA-Bound Ultrabithorax-Extradenticle Homeodomain Complex. Nature 397, 714–719. 10.1038/17833 10067897

[B156] Patterson L. T. Pembaur M. Potter S. S. (2001). Hoxa11andHoxd11regulate Branching Morphogenesis of the Ureteric Bud in the Developing Kidney. Development 128, 2153–2161. 10.1242/dev.128.11.2153 11493536

[B157] Paul D. S. Soranzo N. Beck S. (2014). Functional Interpretation of Non‐coding Sequence Variation: Concepts and Challenges. Bioessays 36, 191–199. 10.1002/bies.201300126 24311363 PMC3992842

[B158] Pearson J. C. Lemons D. Mcginnis W. (2005). Modulating Hox Gene Functions during Animal Body Patterning. Nat. Rev. Genet. 6, 893–904. 10.1038/nrg1726 16341070

[B159] Penkov D. San Martín D. M. Fernandez-Díaz L. C. Rosselló C. A. Torroja C. Sánchez-Cabo F. (2013). Analysis of the DNA-Binding Profile and Function of TALE Homeoproteins Reveals Their Specialization and Specific Interactions with Hox Genes/proteins. Cel Rep. 3, 1321–1333. 10.1016/j.celrep.2013.03.029 23602564

[B160] Perry G. H. Dominy N. J. Claw K. G. Lee A. S. Fiegler H. Redon R. (2007). Diet and the Evolution of Human Amylase Gene Copy Number Variation. Nat. Genet. 39, 1256–1260. 10.1038/ng2123 17828263 PMC2377015

[B161] Phelan M. L. Rambaldi I. Featherstone M. S. (1995). Cooperative Interactions between HOX and PBX Proteins Mediated by a Conserved Peptide Motif. Mol. Cel Biol 15, 3989–3997. 10.1128/mcb.15.8.3989 PMC230638 7623795

[B162] Phelan M. L. Sadoul R. Featherstone M. S. (1994). Functional Differences between HOX Proteins Conferred by Two Residues in the Homeodomain N-Terminal Arm. Mol. Cel. Biol. 14, 5066–5075. 10.1128/mcb.14.8.5066 PMC359025 7913516

[B163] Piper D. E. Batchelor A. H. Chang C.-P. Cleary M. L. Wolberger C. (1999). Structure of a HoxB1-Pbx1 Heterodimer Bound to DNA. Cell 96, 587–597. 10.1016/s0092-8674(00)80662-5 10052460

[B164] Ponting C. P. Russell R. R. (2002). The Natural History of Protein Domains. Annu. Rev. Biophys. Biomol. Struct. 31, 45–71. 10.1146/annurev.biophys.31.082901.134314 11988462

[B165] Pöpperl H. Bienz M. Studer M. Chan S.-K. Aparicio S. Brenner S. (1995). Segmental Expression of Hoxb-1 Is Controlled by a Highly Conserved Autoregulatory Loop Dependent upon Exd/pbx. Cell 81, 1031–1042. 10.1016/s0092-8674(05)80008-x 7600572

[B166] Quiring R. Walldorf U. Kloter U. Gehring W. J. (1994). Homology of the Eyeless Gene of Drosophila to the Small Eye Gene in Mice and Aniridia in Humans. Science 265, 785–789. 10.1126/science.7914031 7914031

[B167] Reilly S. K. Noonan J. P. (2016). Evolution of Gene Regulation in Humans. Annu. Rev. Genom. Hum. Genet. 17, 45–67. 10.1146/annurev-genom-090314-045935 27147089

[B168] Remacle S. Abbas L. De Backer O. Pacico N. Gavalas A. Gofflot F. (2004). Loss of Function but No Gain of Function Caused by Amino Acid Substitutions in the Hexapeptide of Hoxa1 *In Vivo* . Mol. Cel Biol 24, 8567–8575. 10.1128/mcb.24.19.8567-8575.2004 PMC516739 15367676

[B169] Richter D. J. Fozouni P. Eisen M. B. King N. (2018). Gene Family Innovation, Conservation and Loss on the Animal Stem Lineage. Elife 7, 34226. 10.7554/eLife.34226 PMC6040629 29848444

[B170] Rivas M. L. Espinosa-Vázquez J. M. Sambrani N. Greig S. Merabet S. Graba Y. (2013). Antagonism versus Cooperativity with TALE Cofactors at the Base of the Functional Diversification of Hox Protein Function. Plos Genet. 9, e1003252. 10.1371/journal.pgen.1003252 23408901 PMC3567137

[B171] Roberts Kingman G. A. Lee D. Jones F. C. Desmet D. Bell M. A. Kingsley D. M. (2021a). Longer or Shorter Spines: Reciprocal Trait Evolution in Stickleback via Triallelic Regulatory Changes in Stanniocalcin2a. Proc. Natl. Acad. Sci. U S A. 118, 694118. 10.1073/pnas.2100694118 PMC8346906 34321354

[B172] Roberts Kingman G. A. Vyas D. N. Jones F. C. Brady S. D. Chen H. I. Reid K. (2021b). Predicting Future from Past: The Genomic Basis of Recurrent and Rapid Stickleback Evolution. Sci. Adv. 7, 5285. 10.1126/sciadv.abg5285 PMC8213234 34144992

[B173] Rogers J. Gibbs R. A. (2014). Comparative Primate Genomics: Emerging Patterns of Genome Content and Dynamics. Nat. Rev. Genet. 15, 347–359. 10.1038/nrg3707 24709753 PMC4113315

[B174] Ronshaugen M. Mcginnis N. Mcginnis W. (2002). Hox Protein Mutation and Macroevolution of the Insect Body Plan. Nature 415, 914. 10.1038/nature716 11859370

[B175] Rubinstein M. De Souza F. S. J. (2013). Evolution of Transcriptional Enhancers and Animal Diversity. Phil. Trans. R. Soc. B 368, 20130017. 10.1098/rstb.2013.0017 24218630 PMC3826491

[B176] Ryoo H. D. Marty T. Casares F. Affolter M. Mann R. S. (1999). Regulation of Hox Target Genes by a DNA Bound Homothorax/Hox/Extradenticle Complex. Development 126, 5137–5148. 10.1242/dev.126.22.5137 10529430

[B177] Saadaoui M. Merabet S. Litim-Mecheri I. Arbeille E. Sambrani N. Damen W. (2011). Selection of Distinct Hox-Extradenticle Interaction Modes fine-tunes Hox Protein Activity. Proc. Natl. Acad. Sci. U.S.A. 108, 2276–2281. 10.1073/pnas.1006964108 21262810 PMC3038764

[B178] Sakazume S. Sorokina E. Iwamoto Y. Semina E. V. (2007). Functional Analysis of Human Mutations in Homeodomain Transcription Factor PITX3. BMC Mol. Biol. 8, 84. 10.1186/1471-2199-8-84 17888164 PMC2093940

[B179] Sánchez-Higueras C. Sotillos S. Castelli-Gair Hombría J. (2014). Common Origin of Insect Trachea and Endocrine Organs from a Segmentally Repeated Precursor. Curr. Biol. 24, 76–81. 10.1016/j.cub.2013.11.010 24332544

[B180] Sandve S. R. Rohlfs R. V. Hvidsten T. R. (2018). Subfunctionalization versus Neofunctionalization after Whole-Genome Duplication. Nat. Genet. 50, 908–909. 10.1038/s41588-018-0162-4 29955176

[B181] Saurin A. J. Delfini M. C. Maurel-Zaffran C. Graba Y. (2018). The Generic Facet of Hox Protein Function. Trends Genet. 34, 941–953. 10.1016/j.tig.2018.08.006 30241969

[B182] Scannell D. R. Byrne K. P. Gordon J. L. Wong S. Wolfe K. H. (2006). Multiple Rounds of Speciation Associated with Reciprocal Gene Loss in Polyploid Yeasts. Nature 440, 341–345. 10.1038/nature04562 16541074

[B183] Schneuwly S. Klemenz R. Gehring W. J. (1987). Redesigning the Body Plan of Drosophila by Ectopic Expression of the Homoeotic Gene Antennapedia. Nature 325, 816–818. 10.1038/325816a0 3821869

[B184] Scott M. P. Tamkun J. W. Hartzell III G. W. (1989). 3dThe Structure and Function of the Homeodomain. Biochim. Biophys. Acta (Bba) - Rev. Cancer 989, 25–48. 10.1016/0304-419x(89)90033-4 2568852

[B185] Sekigami Y. Kobayashi T. Omi A. Nishitsuji K. Ikuta T. Fujiyama A. (2017). Hox Gene Cluster of the Ascidian, Halocynthia Roretzi, Reveals Multiple Ancient Steps of Cluster Disintegration during Ascidian Evolution. Zoolog. Lett 3, 17. 10.1186/s40851-017-0078-3 PMC5602962 28932414

[B186] Seo H.-C. Edvardsen R. B. Maeland A. D. Bjordal M. Jensen M. F. Hansen A. (2004). Hox Cluster Disintegration with Persistent Anteroposterior Order of Expression in *Oikopleura dioica* . Nature 431, 67–71. 10.1038/nature02709 15343333

[B187] Session A. M. Uno Y. Kwon T. Chapman J. A. Toyoda A. Takahashi S. (2016). Genome Evolution in the Allotetraploid Frog *Xenopus laevis* . Nature 538, 336–343. 10.1038/nature19840 27762356 PMC5313049

[B188] Shanmugam K. Green N. C. Rambaldi I. Saragovi H. U. Featherstone M. S. (1999). PBX and MEIS as Non-DNA-binding Partners in Trimeric Complexes with HOX Proteins. Mol. Cel Biol 19, 7577–7588. 10.1128/mcb.19.11.7577 PMC84774 10523646

[B189] Shen W.-F. Rozenfeld S. Lawrence H. J. Largman C. (1997b). The Abd-B-like Hox Homeodomain Proteins Can Be Subdivided by the Ability to Form Complexes with Pbx1a on a Novel DNA Target. J. Biol. Chem. 272, 8198–8206. 10.1074/jbc.272.13.8198 9079637

[B190] Shen W. F. Montgomery J. C. Rozenfeld S. Moskow J. J. Lawrence H. J. Buchberg A. M. (1997a). AbdB-like Hox Proteins Stabilize DNA Binding by the Meis1 Homeodomain Proteins. Mol. Cel Biol 17, 6448–6458. 10.1128/mcb.17.11.6448 PMC232497 9343407

[B191] Shoubridge C. Tan M. H. Seiboth G. Gecz J. (2012). ARX Homeodomain Mutations Abolish DNA Binding and lead to a Loss of Transcriptional Repression. Hum. Mol. Genet. 21, 1639–1647. 10.1093/hmg/ddr601 22194193

[B192] Singh N. P. De Kumar B. Paulson A. Parrish M. E. Scott C. Zhang Y. (2021). Genome-Wide Binding Analyses of HOXB1 Revealed a Novel DNA Binding Motif Associated with Gene Repression. J. Dev. Biol. 9, 6. 10.3390/jdb9010006 33546292 PMC7931043

[B193] Singh N. P. De Kumar B. Paulson A. Parrish M. E. Zhang Y. Florens L. (2020). A Six-Amino-Acid Motif Is a Major Determinant in Functional Evolution of HOX1 Proteins. Genes Dev. 34, 1680–1696. 10.1101/gad.342329.120 33184220 PMC7706710

[B194] Slattery M. Riley T. Liu P. Abe N. Gomez-Alcala P. Dror I. (2011). Cofactor Binding Evokes Latent Differences in DNA Binding Specificity between Hox Proteins. Cell 147, 1270–1282. 10.1016/j.cell.2011.10.053 22153072 PMC3319069

[B195] Smith J. J. Timoshevskaya N. Ye C. Holt C. Keinath M. C. Parker H. J. (2018). The Sea Lamprey Germline Genome Provides Insights into Programmed Genome Rearrangement and Vertebrate Evolution. Nat. Genet. 50, 270–277. 10.1038/s41588-017-0036-1 29358652 PMC5805609

[B196] Sorge S. Ha N. Polychronidou M. Friedrich J. Bezdan D. Kaspar P. (2012). Thecis-regulatory Code of Hox Function inDrosophila. EMBO J. 31, 3323–3333. 10.1038/emboj.2012.179 22781127 PMC3411081

[B197] Soshnikova N. Dewaele R. Janvier P. Krumlauf R. Duboule D. (2013). Duplications of Hox Gene Clusters and the Emergence of Vertebrates. Develop. Biol. 378, 194–199. 10.1016/j.ydbio.2013.03.004 23501471

[B198] Spitz F. Gonzalez F. Peichel C. Vogt T. F. Duboule D. Zákány J. (2001). Large Scale Transgenic and Cluster Deletion Analysis of the HoxD Complex Separate an Ancestral Regulatory Module from Evolutionary Innovations. Genes Dev. 15, 2209–2214. 10.1101/gad.205701 11544178 PMC312772

[B199] Suemori H. Noguchi S. (2000). Hox C Cluster Genes Are Dispensable for Overall Body Plan of Mouse Embryonic Development. Develop. Biol. 220, 333–342. 10.1006/dbio.2000.9651 10753520

[B200] Truong D. M. Boeke J. D. (2017). Resetting the Yeast Epigenome with Human Nucleosomes. Cell 171, 1508–1519. 10.1016/j.cell.2017.10.043 29198523 PMC5732057

[B201] Tvrdik P. Capecchi M. R. (2006). Reversal of Hox1 Gene Subfunctionalization in the Mouse. Develop. Cel 11, 239–250. 10.1016/j.devcel.2006.06.016 16890163

[B202] Van Den Akker E. Fromental-Ramain C. De Graaff W. Le Mouellic H. Brulet P. Chambon P. (2001). Axial Skeletal Patterning in Mice Lacking All Paralogous Group 8 Hox Genes. Development 128, 1911–1921. 10.1242/dev.128.10.1911 11311170

[B203] Van Dijk M. A. Murre C. (1994). *Extradenticle* Raises the DNA Binding Specificity of Homeotic Selector Gene Products. Cell 78, 617–624. 10.1016/0092-8674(94)90526-6 7915200

[B204] Vandenbussche M. Theissen G. Van De Peer Y. Gerats T. (2003). Structural Diversification and Neo-Functionalization during floral MADS-Box Gene Evolution by C-Terminal Frameshift Mutations. Nucleic Acids Res. 31, 4401–4409. 10.1093/nar/gkg642 12888499 PMC169922

[B205] Vandepoele K. De Vos W. Taylor J. S. Meyer A. Van De Peer Y. (2004). Major Events in the Genome Evolution of Vertebrates: Paranome Age and Size Differ Considerably between ray-finned Fishes and Land Vertebrates. Proc. Natl. Acad. Sci. U.S.A. 101, 1638–1643. 10.1073/pnas.0307968100 14757817 PMC341801

[B206] Vieux-Rochas M. Mascrez B. Krumlauf R. Duboule D. (2013). Combined Function of HoxA and HoxB Clusters in Neural Crest Cells. Develop. Biol. 382, 293–301. 10.1016/j.ydbio.2013.06.027 23850771

[B207] Wagner G. P. Amemiya C. Ruddle F. (2003). Hox Cluster Duplications and the Opportunity for Evolutionary Novelties. Proc. Natl. Acad. Sci. U.S.A. 100, 14603–14606. 10.1073/pnas.2536656100 14638945 PMC299744

[B208] Wahba G. M. Hostikka S. L. Carpenter E. M. (2001). The Paralogous Hox Genes Hoxa10 and Hoxd10 Interact to Pattern the Mouse Hindlimb Peripheral Nervous System and Skeleton. Develop. Biol. 231, 87–102. 10.1006/dbio.2000.0130 11180954

[B209] Walsh B. (2003). Population-genetic Models of the Fates of Duplicate Genes. Genetica 118, 279–294. 10.1007/978-94-010-0229-5_16 12868616

[B210] Webb B. D. Shaaban S. Gaspar H. Cunha L. F. Schubert C. R. Hao K. (2012). HOXB1 Founder Mutation in Humans Recapitulates the Phenotype of Hoxb1 Mice. Am. J. Hum. Genet. 91, 171–179. 10.1016/j.ajhg.2012.05.018 22770981 PMC3397264

[B211] Wendel J. F. (2000). Genome Evolution in Polyploids. Plant Mol. Biol. 42, 225–249. 10.1007/978-94-011-4221-2_12 10688139

[B212] Xie K. T. Wang G. Thompson A. C. Wucherpfennig J. I. Reimchen T. E. Maccoll A. D. C. (2019). DNA Fragility in the Parallel Evolution of Pelvic Reduction in Stickleback Fish. Science 363, 81–84. 10.1126/science.aan1425 30606845 PMC6677656

[B213] Yokouchi Y. Nakazato S. Yamamoto M. Goto Y. Kameda T. Iba H. (1995). Misexpression of *Hoxa-13* Induces Cartilage Homeotic Transformation and Changes Cell Adhesiveness in Chick Limb Buds. Genes Dev. 9, 2509–2522. 10.1101/gad.9.20.2509 7590231

[B214] Young T. Rowland J. E. Van De Ven C. Bialecka M. Novoa A. Carapuco M. (2009). Cdx and Hox Genes Differentially Regulate Posterior Axial Growth in Mammalian Embryos. Develop. Cel 17, 516–526. 10.1016/j.devcel.2009.08.010 19853565

[B215] Zappavigna V. Sartori D. Mavilio F. (1994). Specificity of HOX Protein Function Depends on DNA-Protein and Protein-Protein Interactions, Both Mediated by the Homeo Domain. Genes Dev. 8, 732–744. 10.1101/gad.8.6.732 7926763

[B216] Zhao J. J. Lazzarini R. A. Pick L. (1993). The Mouse Hox-1.3 Gene Is Functionally Equivalent to the Drosophila Sex combs Reduced Gene. Genes Dev. 7, 343–354. 10.1101/gad.7.3.343 8095481

[B217] Zhao Y. Potter S. S. (2002). Functional Comparison of the Hoxa 4, Hoxa 10, and Hoxa 11 Homeoboxes. Develop. Biol. 244, 21–36. 10.1006/dbio.2002.0595 11900456

[B218] Zhao Y. Potter S. S. (2001). Functional Specificity of theHoxa13homeobox. Development 128, 3197–3207. 10.1242/dev.128.16.3197 11688568

[B219] Zhen Y. Andolfatto P. (2012). Methods to Detect Selection on Noncoding DNA. Methods Mol. Biol. 856, 141–159. 10.1007/978-1-61779-585-5_6 22399458 PMC3725466

[B220] Zimmer C. T. Garrood W. T. Singh K. S. Randall E. Lueke B. Gutbrod O. (2018). Neofunctionalization of Duplicated P450 Genes Drives the Evolution of Insecticide Resistance in the Brown Planthopper. Curr. Biol. 28, 268–274. 10.1016/j.cub.2017.11.060 29337073 PMC5788746

